# Palm tree disease detection and classification using residual network and transfer learning of inception ResNet

**DOI:** 10.1371/journal.pone.0282250

**Published:** 2023-03-02

**Authors:** Mostafa Ahmed, Ali Ahmed

**Affiliations:** 1 Computer Science Department, Faculty of Computers and Information, Menoufia University, Shibin El Kom, Egypt; 2 Information Technology Department, Faculty of Computers and Information, Menoufia University, Shibin El Kom, Egypt; Zonguldak Bülent Ecevit University: Zonguldak Bulent Ecevit Universitesi, TURKEY

## Abstract

Agriculture has become an essential field of study and is considered a challenge for many researchers in computer vision specialization. The early detection and classification of plant diseases are crucial for preventing growing diseases and hence yield reduction. Although many state-of-the-artwork proposed various classification techniques for plant diseases, still face many challenges such as noise reduction, extracting the relevant features, and excluding the redundant ones. Recently, deep learning models are noticeable as hot research and are widely used for plant leaf disease classification. Although the achievement with these models is notable, still the need for efficient, fast-trained, and few-parameters models without compromising on performance is inevitable. In this work, two approaches of deep learning have been proposed for Palm leaf disease classification: Residual Network (ResNet) and transfer learning of Inception ResNet. The models make it possible to train up to hundreds of layers and achieve superior performance. Considering the merit of their effective representation ability, the performance of image classification using ResNet has been boosted, such as diseases of plant leaves classification. In both approaches, problems such as variation of luminance and background, different scales of images, and inter-class similarity have been treated. Date Palm dataset having 2631 colored images with varied sizes was used to train and test the models. Using some well-known metrics, the proposed models outperformed many of the recent research in the field in original and augmented datasets and achieved an accuracy of 99.62% and 100% respectively.

## I. Introduction

In the Middle East Area since early times Palms and their fruits have been considered one of the most important crops as well as an essential part of many cultures. One of the important features of palms is that they are evergreen plants as well as their leaves of various ages are present continuously on palm trees throughout the year. So, they are considered hosts of many insects, and they are reliable sources of food. Young and old foliage of palms is preferred for different kinds of insect species. Because the size and the number of fronds in the crown basically stay the same as soon as the crown is fully formed, the amount of food available does not change materially.

Among the many varieties of insect species, the red palm weevil (RPW) that can affect coconut palms is considered the most critical pest for palms and was a problem in many countries for a very long time, especially in Gulf region since the 80s. The oil palm yields can be increased by taking care of the health of the palm trees and by early preventing and treating several diseases infections such as fungi, bacteria and, viruses [1]. Because palm trees are evergreen plants, oil palm trees can be infected by several diseases during all their growing stages [2]. Although much researchers’ effort, still there are no satisfactory methods that can be relied on to prevent the reduction in yield due to disease infection. Palm tree infections usually have remarkable symptoms and the traditional method to decrease the infection is by visual inspection which is expensive as it uses an intensive labor force and consumes much time as well as more probable for misclassification of the disease [3, 4].

Recently, among the many studies in plant disease classification, some have focused on machine learning as a powerful tool. Plant disease classification using machine learning methods mainly consists of three stages: first, remove the background or segment the infected part using preprocessing techniques; second, extract distinguishing features for further classification analysis; finally, supervised or unsupervised algorithms are used to classify the selected features. A convolution net (CNN) is one of the important deep learning algorithms and is the most widely used algorithm for plant leaf disease classification. CNN mainly includes convolutional layers, pooling layers, and fully connected layers. The convolutional layer extracts feature from the local correlation of the information in the image. For the classification task, many CNN-based classification models have been developed in deep learning-related research such as AlexNet, VGGNet, GoogLeNet, ResNet, MobileNet, and EfficientNet. Recently, it was concluded by researchers in deep learning that when the number of deep CNN layers reached a certain depth, it would cause the network to converge more slowly and not improve the classification performance. ResNet network was developed by Microsoft lab in 2015 to solve the problem of gradient elimination or gradient explosion by proposing residual blocks and shortcut connections. ResNet influenced the development direction of deep learning in academic and industry research. The problem of detecting and classifying fruit and vegetable disease using deep learning models by many researchers is reported in the literature. In this section, we will briefly introduce many of the researchers’ contributions related to plant diseases. In [5], an approach is proposed for detecting palm tree diseases using CNN and SVM classifiers. In this research, CNN is used to differentiate between the two diseases named leaf and blight spots, and SVM is used for detecting Red Palm Weevil (RPW) pests. The accuracy ratios of the two classifiers are 97.9% and 92.8% respectively. Magsi et al. present a method for identifying (Sudden Decline Syndrome) disease identification at all the date palms infection stages using CNN and they obtained a success accuracy rate of 89.4% [6].

The authors in [7] proposed a modified MobileNetV2 neural network to improve the accuracy rate of cassava leaf disease on lower-quality testing images utilizing data augmentation techniques. They obtained a recognition accuracy of 97.7% when they used high-quality images and a significantly lower accuracy with low-quality images. For of guava plant diseases classification, the authors in [8] used a batch of classifiers such as Fine KNN, Cubic SVM, Complex tree, Boosted tree, and Bagged tree ensemble for image-level and they obtained an overall classification accuracy of 99%. A hybrid model of wrapper approach consists of FPA-SVM and CNN classifiers is proposed for plant diseases classification [9]. In this approach, the features were selected with a wrapper approach consisting of FPA and SVM to keep the classifier performance high and an accuracy of 99.6% was achieved. The authors in [10] proposed a deep learning model based on an optimization algorithm for recognizing cucumber and potato leaf diseases. They extracted deep features from the global pooling layer in the next step tuned using enhanced Cuckoo search algorithm and they achieved an accuracy of 99.2%. Atila et. al proposed EfficientNet DL architecture for classifying plant leaf diseases [11]. Their model and other DL models were trained using transfer learning approach and they achieved accuracy of 99.91% and 99.97% when tested using original and augmented datasets respectively. Rehman et al. proposed a new deep learning model for classifying Citrus disease [12]. In their study, two pre-trained MobileNetv2 and DenseNet201 models are retrained using transfer learning to obtain feature vectors optimized using the Whale Optimization Algorithm (WOA) and they achieved an accuracy rate of 95.7%. For guava disease identification, a DL model with augmented enhanced data using color-histogram equalization and unsharp masking technique is proposed and achieved accuracy rate of 97.7% [13].

For weed detection in soybean plantations, five deep learning models consists of MobileNetV2, ResNet50, and three proposed models are used by the authors in [14], and they achieved an accuracy of 97.7%. In [15], the authors proposed a framework for recognizing cucumber leaf diseases using pre-trained deep CNN models. A dataset for cucumber leaf was augmented for growing dataset images and was used to test the performance of the implemented models and an accuracy of 98.48% was obtained. For the classification of Marsonina Coronaria and Apple Scab diseases from apple leaves, the authors in [16] proposed a CNN model with 19 convolutional layers for accurately detecting the disease and they achieved accuracy of 99.2%. A real-time detector based on GLDD and Faster R-CNN detection algorithm for grape leaf diseases is proposed in [17]. In this research, a faster DR-IACNN model with higher feature extraction capability is introduced for detection by proposing the Inception-v1, Inception-ResNet-v2, and SE-blocks. In [18], cotton leaf disease spots features were extracted and classified using CNN and an accuracy of 95.13% was obtained. The authors in [19], used the deep learning models named (SVM, NN, GoogLeNet, and VGG16) to recognize and classify Wheat diseases leaves and achieved 98%. A comparative study using deep learning models for identifying plant disease was introduced in [20]. In this study, the tuned implemented models were VGG 16, Inception V4, ResNet with 50, 101 and 152 layers and DenseNets with 121 layers and are utilized for classifying 26 diseases of 14 different plants. Recent work for palm tree disease classification using a convolution neural network (CNN) was proposed in [21]. In this work, the model was used for the detection and classification of the four well-known diseases threatening palms recently such as Bacterial leaf blight, Brown spots, Leaf smut, white scale as well as the healthy leaves. The performance of the proposed model was tested against two well-known CNN models called VGG-16 and MobileNet and an accuracy rate of 99.23% was achieved. Another study of palm tree disease detection and classification was done by Hamdani et. al [22]. In this study, the authors generated color features using principal component analysis (PCA), classified the palm diseases using ANN classifier, and they achieved accuracy of 99.67%. Some recent research in plant diseases classification is summarized in Table 1.

**Table 1:** Results of previous work using deep learning for identification of plant diseases.

Ref. No.	Date	DL model	Species	Metrics	Disease/infection	Accuracy rate
5	2020	CNN	date palm	Precision and F1 Score	Red Palm Weevil	97.9%
6	2020	CNN	date palm	Recognition accuracy	Sudden Decline Syndrome	95.6%
7	2021	modified MobileNetV2	cassava	Recognition accuracy	Bacterial blight, green mite damage, and streak virus disease	97.7%
8	2021	Fine KNN, Cubic SVM, Complex tree, Boosted tree, and Bagged tree	Guava	Sensitivity, Specificity, and accuracy	Canker, Mummification, Dot, Rust	99%
9	2022	FPA-SVM and PSO-SVM	apple, grape, and tomato	Recall, precision, and F1 Score	black rot, cedar rust, and scab	99.6%
10	2023	DarkNet19	Potato, Tomato, Cucumber	Sensitivity, Precision, F1 score and accuracy	anthracnose, downy mildew, powdery mildew, and target leaf spots	99.2%
11	2021	EfficientNet	Different plants	Sensitivity, Specificity, Precision, and accuracy	bacterial spot, rust	99.91%
12	2022	MobileNetv2 and DenseNet201	Citrus	Sensitivity, Specificity, Precision, F1 score and accuracy	Different citrus diseases	95.7%
13	2021	AlexNet, SqueezeNet, GoogLeNet, ResNet-50, and ResNet-101	Guava	Sensitivity, Specificity, Precision, F1 score and accuracy	fungal diseases	97.74%
14	2022	MobileNetV2, ResNet50	Soybean	Recognition accuracy	Weeds	97.7%
15	2022	Deep Entropy-ELM	Cucumber	Recall, precision, and F1 Score	anthracnose, powdery mildew, downy mildew, and cucumber mosaic c	98.48%
16	2022	CNN	Apple	Accuracy, Sensitivity and Specificity	Marsonina Coronaria and Apple Scab	99.2%
17	2020	CNN	Grape	Precision	Black rot, Black measles, Leaf blight and Mites	81.1%
18	2021	CNN	Cotton	Recognition Accuracy	disease spot	91.75%
19	2020	SVM, NN, GoogLeNet, and VGG16	Wheat	Recognition Accuracy	tan spot and leaf rust	98%
20	2019	VGG 16, Inception V4, ResNet	14 different plants	Recognition Accuracy	26 diseases	99.75%
21	2022	CNN	Palm tree	Accuracy, Precision, Recall and F1 Score	Bacterial leaf blight, Brown spots, Leaf smut, white scale	99.23%
22	2021	ANN	Palm tree	sensitivity, specificity, and accuracy	curvularia leaf spots	99.67%

Although the many previous state-of-art studies concerning the use of deep learning models on the detection and classification of plant leaf diseases, still there are gaps to be addressed concerning the use of these models in palm leaf disease detection and classification. In particular, the need for efficient, fast-trained, and few-parameters models without compromising on performance is inevitable. To overcome the above-mentioned problems, three deep learning models are proposed for Palm leaf diseases classification called Residual Network (ResNet) based on [23], Modified Residual Network (MResNet), and Inception Residual Network (IncResNet) based on [24]. The main contributions of this study can be summarized in a few points:

- Implementing a deep learning model by using ResNet for classifying palm leaf diseases.
- Implementing a modified ResNet Model (MResNet model) by adapting the filter sizes to effectively detect and classify palm leaf diseases.
- Implementing a transfer learning model by using ResNet (IncResNet model) for classifying palm leaf diseases.
- Study the effect of different numbers of epochs as a hyperparameter in the training process.
- Providing comparative analytics of the implemented models with some of the state-of-art models of classifying plant disease.

In this research, the work is organized as follows: Section one introduces and summarizes the literature review; Section two presents the methodology used in this research, Section three discusses in detail the results and the comparison of the proposed approach with some of the well-known deep learning models, and finally, Section four gives the conclusion for the proposed study and the suggestion for future research.

## II. Methodology

**Fig 1:**
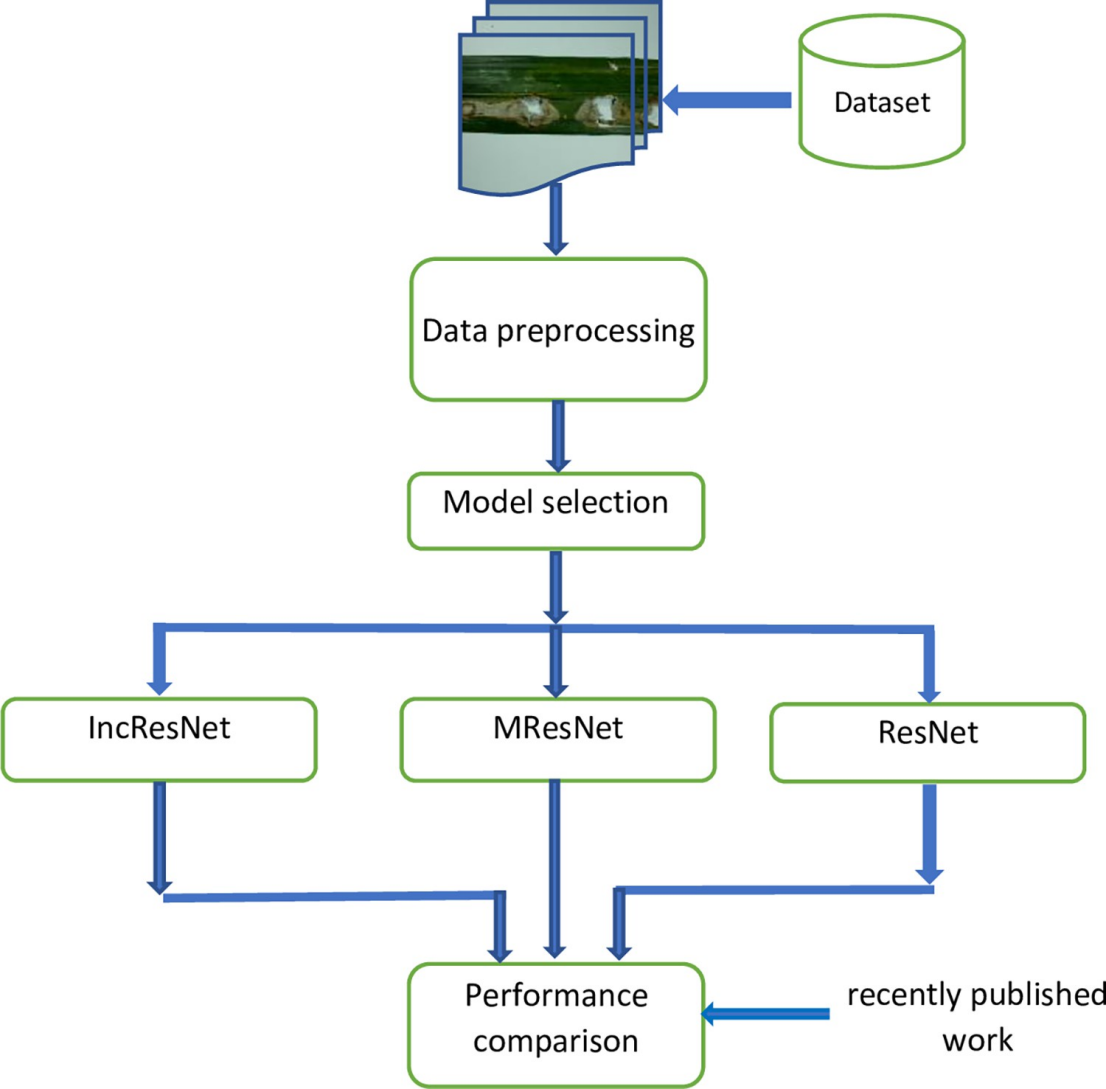
The proposed palm tree disease identification using deep learning models.

The proposed palm leaf diseases identification framework is illustrated in Fig 1. The proposed framework includes the following important stages. First, the data is augmented to increase the number of samples used for training process. Next, three pre-trained deep learning models are selected and fine-tuned. These models are then trained using transfer learning and deep features are extracted from the global pooling layer. Finally, comparisons with well-known and recent deep leaning models are done.

In this research, we implement deep neural network models with residual connections to train on the Palm-leaves dataset. Residual connections are considered an effective way in deep neural network architectures to improve gradient flow through the network and ease the training process of it. Simple neural networks that consist of a simple sequence of layers are sufficient for many applications. However, the more complex problems require more complex network architectures that have multiple layers to send inputs to multiple layers and to receive outputs. Some of these types of network architectures are often called directed acyclic graph (DAG) structured-based networks. The residual network is one of a DAG network that has a skip or residual connections between its layers. The residual connections can skip the main network layers for enabling the propagation of parameter gradients more smoothly from the output layer to the earlier layers. This enhances network performance and accuracy while making it possible to train networks more easily. With increasing the depth of the network layers, higher accuracies can be achieved to accomplish difficult tasks such as differentiating between the leaves of Palm Date that contain a very small, infected area of diseases. The three implemented models will be discussed on the following subsections.

### A) Data augmentation

Data augmentation was frequently used to cover model overfitting problems require a large amount of data for training and hence improving model prediction accuracy. Such model relies on a large amount of data to overcome the problem of overfitting. Due to overfitting, the model is unable to accurately predict cases other than training data. Data augmentation methods can enable machine learning models to be more efficient by creating variations that the model can predict in the real world. In addition, data augmentation was performed using transformation methods to enhance the region of interest (ROI) and to remove any existing noise. The final augmented and improved data are then fed into the fine-tuned implemented models.

### B) ResNet architecture

**Fig 2:**
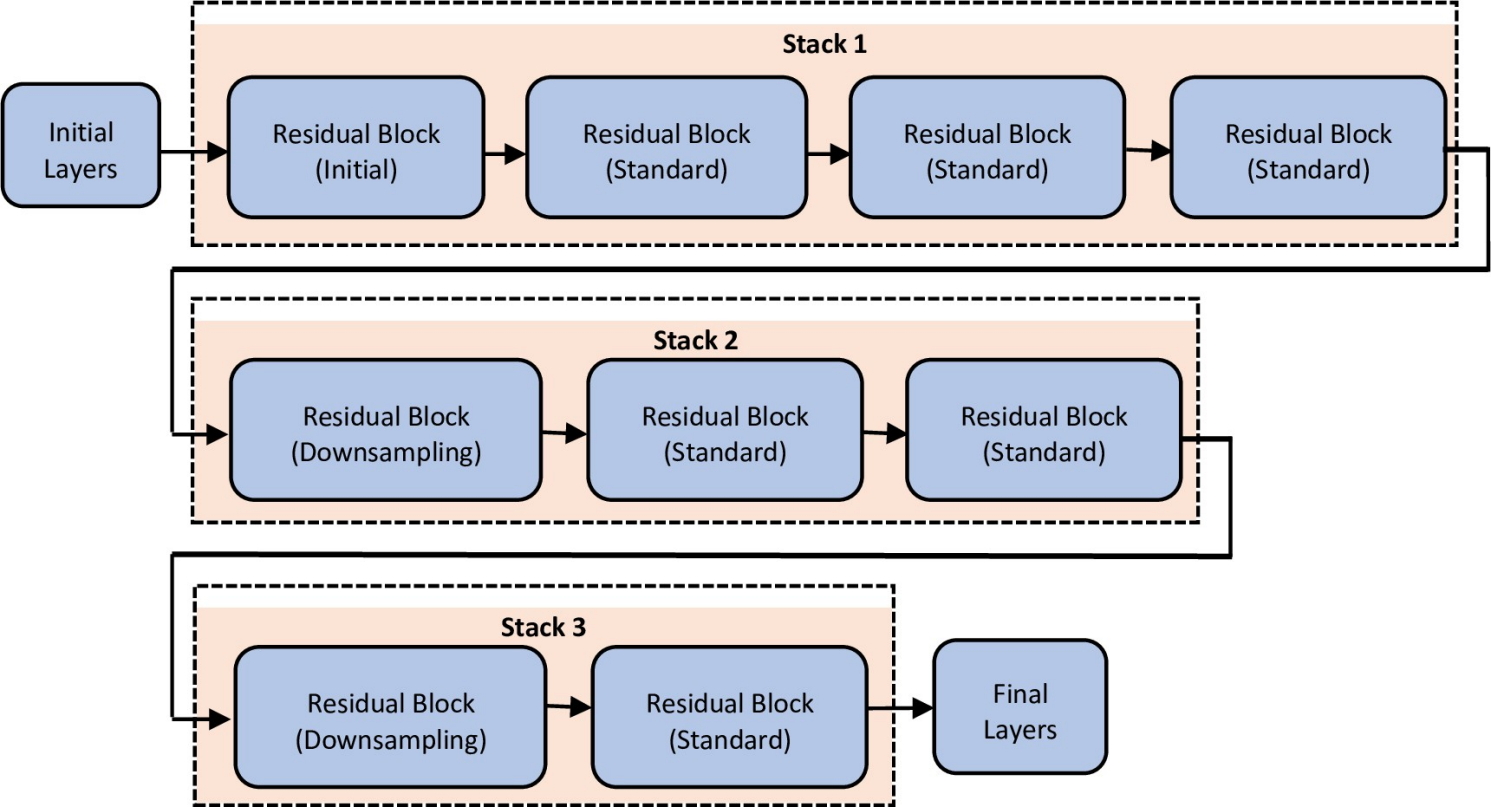
The proposed ResNet architecture.

A ResNet architecture is mainly composed of three types of layers. The first layers are the initial layers, the second are stacks of residual blocks, and the third is the final layers as depicted in Fig 2. Moreover, the residual blocks have also three types named initial residual block, standard residual block, and down-sampling residual block. The initial residual block appears at the beginning of the first stack and uses bottleneck components. So, the initial residual layers resample the down sampling block layers just with a stride of [1,1] in the first convolutional layer. The standard residual block appears more than once in each stack and keeps the activation sizes. The down-sampling residual block appears once at the beginning of each stack except the first one. The spatial dimensions are down sampled by a factor of two by the first convolutional unit of the down-sampling block.

The depth of each stack can change from network to network. In our implementation as seen in Table 2, the residual network has three stacks of depth 4, 3, and 2 respectively. Each residual block inside each stack contains deep learning layers as shown in Table 2. To create and train a residual network suitable for image classification, we followed these steps:

• A residual network has been created.
• The network has been trained.
• Classification and prediction on Palm-leaves data have been performed.

**Table 2:** The deep learning layers in each stack’s block in the proposed ResNet.

Initial Layers	Stack 1				Stack 2			Stack 3		Final Layers
	Block 1	Block 2	Block 3	Block 4	Block 1	Block 2	Block 3	Block 1	Block 2	
Input Layer	Conv2D	Conv2D	Conv2D	Conv2D	Conv2D	Conv2D	Conv2D	Conv2D	Conv2D	Fully Connected
	(1x1, 16)	(1x1, 64)	(1x1, 16)	(1x1, 64)	(1x1, 64)	(1x1, 128)	(1x1, 128)	(1x1, 128)	(1x1, 256)	
Conv2D (7x7, 16)	BN	BN	BN	BN	BN	BN	BN	BN	BN	Softmax
BN	ReLU	ReLU	ReLU	ReLU	ReLU	ReLU	ReLU	ReLU	ReLU	Classification Output
ReLU	Conv2D	Conv2D	Conv2D	Conv2D	Conv2D	Conv2D	Conv2D	Conv2D	Conv2D	--
	(3x3, 16)	(3x3, 16)	(3x3, 16)	(3x3, 16)	(3x3, 32)	(3x3, 32)	(3x3, 32)	(3x3, 64)	(3x3, 64)	
--	BN	BN	BN	BN	BN	BN	BN	BN	BN	--
--	ReLU	ReLU	ReLU	ReLU	ReLU	ReLU	ReLU	ReLU	ReLU	--
--	Conv2D	Conv2D	Conv2D	Conv2D	Conv2D	Conv2D	Conv2D	Conv2D	Conv2D	--
	(1x1, 16)	(1x1, 64)	(1x1, 16)	(1x1, 64)	(1x1, 32)	(1x1, 32)	(1x1, 32)	(1x1, 64)	(1x1, 64)	
--	BN	BN	BN	BN	BN	BN	BN	BN	BN	--

### C) MResNet

Using 1x1 convolution odd-size kernel filters in the previous model leads to the features extracted would be fine-grained and local, without any knowledge about the neighboring pixels. In the modified model, we replaced the 1x1 filters with 3x3 convolutional filters in all blocks for different stacks. Moreover, rising the size of 3x3 filters to 5x5 in the blocks will assume large local areas from images to extract many useful features. The architecture of the modified models is displayed in Table 3.

**Table 3:** Another deep learning layers in each stack’s block in the proposed MResNet.

Initial Layers	Stack 1				Stack 2			Stack 3		Final Layers
	Block 1	Block 2	Block 3	Block 4	Block 1	Block 2	Block 3	Block 1	Block 2	
Input Layer	Conv2D	Conv2D	Conv2D	Conv2D	Conv2D	Conv2D	Conv2D	Conv2D	Conv2D	Fully Connected
	(3x3, 16)	(3x3, 64)	(3x3, 16)	(3x3, 64)	(3x3, 64)	(3x3, 128)	(3x3, 128)	(3x3, 128)	(3x3, 256)	
Conv2D (7x7, 16)	BN	BN	BN	BN	BN	BN	BN	BN	BN	Softmax
BN	ReLU	ReLU	ReLU	ReLU	ReLU	ReLU	ReLU	ReLU	ReLU	Classification Output
ReLU	Conv2D	Conv2D	Conv2D	Conv2D	Conv2D	Conv2D	Conv2D	Conv2D	Conv2D	--
	(5x5, 16)	(5x5, 16)	(5x5, 16)	(5x5, 16)	(5x5, 32)	(5x5, 32)	(5x5, 32)	(5x5, 64)	(5x5, 64)	
--	BN	BN	BN	BN	BN	BN	BN	BN	BN	--
--	ReLU	ReLU	ReLU	ReLU	ReLU	ReLU	ReLU	ReLU	ReLU	--
--	Conv2D	Conv2D	Conv2D	Conv2D	Conv2D	Conv2D	Conv2D	Conv2D	Conv2D	--
	(3x3, 16)	(3x3, 64)	(3x3, 16)	(3x3, 64)	(3x3, 32)	(3x3, 32)	(3x3, 32)	(3x3, 64)	(3x3, 64)	
--	BN	BN	BN	BN	BN	BN	BN	BN	BN	--

### D) IncResNet

Pretrained networks for image classification have been trained on a huge number of images for classifying images into thousands of different categories. Through the training process, these networks have learned rich features from the training dataset images. The main task of the trained network is to take an image as an input and produce a label for the object in the image. It can compute the probabilities for each object to belong to a specific category.

Transfer learning is a common approach used frequently in recent years in deep learning applications [25]. A pre-trained network can be used as a first step to learning how to classify different categories. Adapting a pre-trained network is usually much easier and faster than designing and training a network from scratch. By using non-randomly initialized weights of the pre-trained network, you may fast transfer learned features of the network to classify different categories, especially when a small number of images are available

The task of the first convolutional layers of the network is to extract image features for the last classification layers to classify images. More accurately, the last two layers named "Fully Connected Layer" and "Classification Output Layer" in the pre-trained IncResNet network, have the information to classify the objects in images to the predicted labels with the evaluation of the probabilities and the loss values. To re-train a pre-trained network to classify new dataset images, we replace the last two layers with new layers fine-tuned to the new dataset.

By replacing the fully connected layer with a new one that matched the number of outputs of our Palm-Leaves classes (3 classes, namely, Healthy, White Scale, and Brown Spots) the network can learn the new features of these classes. The classification layer of the network classifies the output classes. By replacing the classification layer (without class labels) with a new one, the network can classify the new images into new classes. The network can automatically set the new output classes during the training process. Our network is now ready to be re-trained once more on our set of Palm-Leaves images. Moreover, the weights of earlier layers in the inception network will be frozen by setting the learning rates to zero. During the training process, the inception network does not update the parameters of the layers that have been frozen. This way, not many computations are needed for the gradients of the frozen layers, this may significantly speed up the network training process.

## III. Experimental results and discussion

The implementation code runs under the Microsoft Windows 10 Home (x64) operating system. The code is written in Matlab 2021b with specific toolboxes. The device used in the implementation is Alienware Aurora R9 desktop equipped with an Octa-core Intel(R) Core (TM) i9-9900 as well as the base frequency of the device is 3.10 GHz. In addition, the device has 32 GB RAM capacity and 1 TB hard disk drive.

### A) Dataset

Palm disease images in this research are used to train and evaluate the proposed deep learning models for the purpose of disease identification and classification from the images. Date Palm data | Kaggle was used for this study. Date Palm data includes three directories: brown spots (470 files), healthy (1203 files), and white scale (958 files). The total images are 2631 colored images with varied sizes.

### B) Experiment configurations

**Fig 3:**
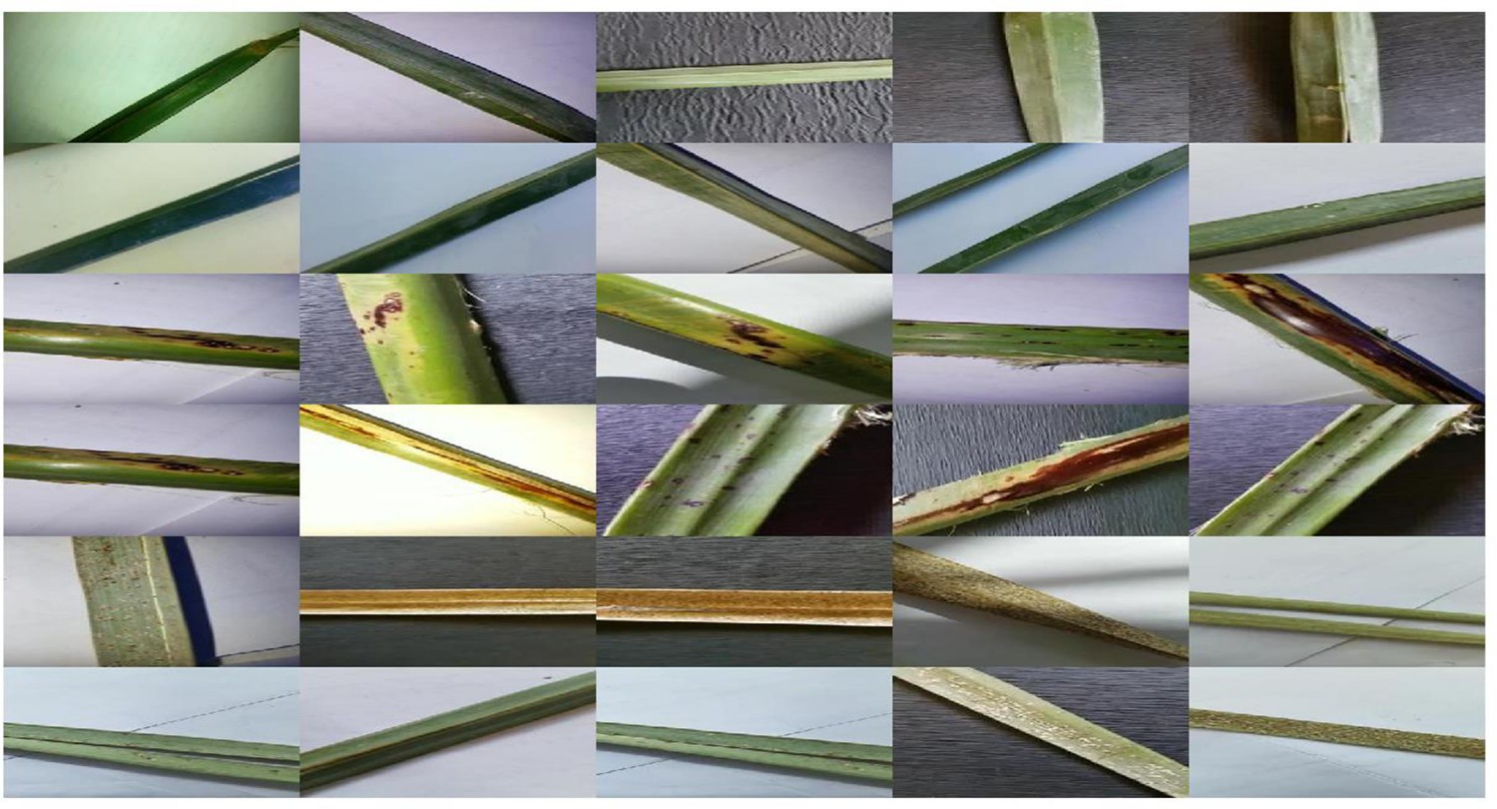
Sample of palm leaves images.

In our experiments, the size of the images is first changed to 192×350×3 for ResNet architectures while for the Inception ResNet architecture, the images are changed to 299×299×3 pixels. Fig 3 shows palm leaf image samples from the dataset used in the experiments. The training data is mainly split into two parts: training and validation data for cross-validation with a ratio of 80% for training and 20% for testing to decide whether the model is overfitting or not. According to the 80/20 rule, overtraining of deep learning networks can be prevented. The performance of the model is checked after each epoch of training by monitoring loss and accuracy on the training set to check the epoch number after which the model starts overfitting. As long as the validation and training error keeps dropping, training should continue.

The activation function Softmax was chosen in the last layer and loss function was selected as categorical cross entropy. The maximum of epochs was defined in the training of models as 30 epochs. Pre-trained models were used with the same optimization method as used on training of Kaggle dataset. All models use stochastic gradient descent with momentum (SGDM) optimizer. In addition, the learning rate was selected as 0.001 for SGDM algorithm. Validation frequency value for all models was set to 210. During the training, using mini-batch, the weights and gradient can be updated after a subset of the total training set is computed by the models. In this work, mini-batch size was set to 10, which was the maximum value allowed by hardware resources in all models. Table 4 shows the hyperparameters used in training both proposed ResNet models as well as the Inception ResNet networks.

**Table 4:** The hyper parameters used in training options for all deep learning ResNet models.

Hyper parameter	Value	Description
Optimizer	SGDM	Stochastic Gradient Descent Momentum solver
Initial Learn Rate	0.001	Global learning rate
Mini Batch Size	10	A subset of the training dataset mainly used to evaluate the gradient of the loss function as well as to update the weights
Max Epochs	[5, 10, 15, 20, 25, 30]	Maximum number of epochs used for training
Validation Frequency	210	Frequency of network validation in number of iterations, specified as a positive integer.
Shuffle	Every Epoch	An option for shuffling data
Learn Rate Schedule	Piecewise	An option for adjusting the learning rate as training progresses
Learn Rate Drop Factor	0.1	Factor for dropping the learning rate every few epochs
Learn Rate Drop Period	60	No. epochs for dropping the learning rate during the training process

### C) Performance evaluation

The seven performance evaluation metrics used for analyzing the models are validation accuracy, training error, validation error, overall precision and recall as well as F1 score. The accuracy metric is defined as:

$$Accuracy=\frac{TP+TN}{T}$$ (1)

In the above equation, *TP* and *TN* are two positive numbers represented the true positive and true negative classifications, respectively, while the positive number *T* denoted the total number of samples. The precision metric can be defined as:

$$Precision=\frac{TP}{TP+FP}$$ (2)

Where the two positive numbers *TP* and *FP* represent the true positive and false positive classifications, respectively. The accuracy metrics Recall on and F1Score can be calculated using precision and recall metrics as follows:

$$Recall=\frac{TP}{TP+FN}$$ (3)

$$F1\;Score=2x\frac{Precision\;x\;Recall}{Precision+Recall}$$ (4)

### D) Proposed ResNet results

**Fig 4:**
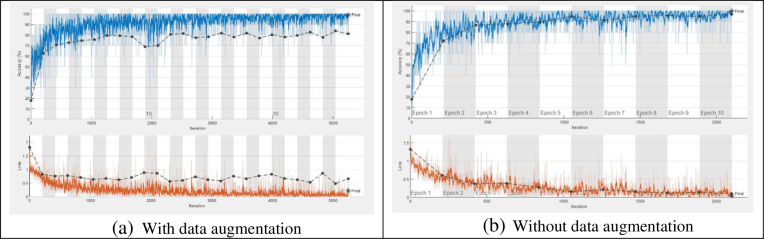
Training progress of the proposed ResNet 1x1, 3x3 with and without data augmentation. (a) With data augmentation; (b) Without data augmentation.

**Fig 5:**
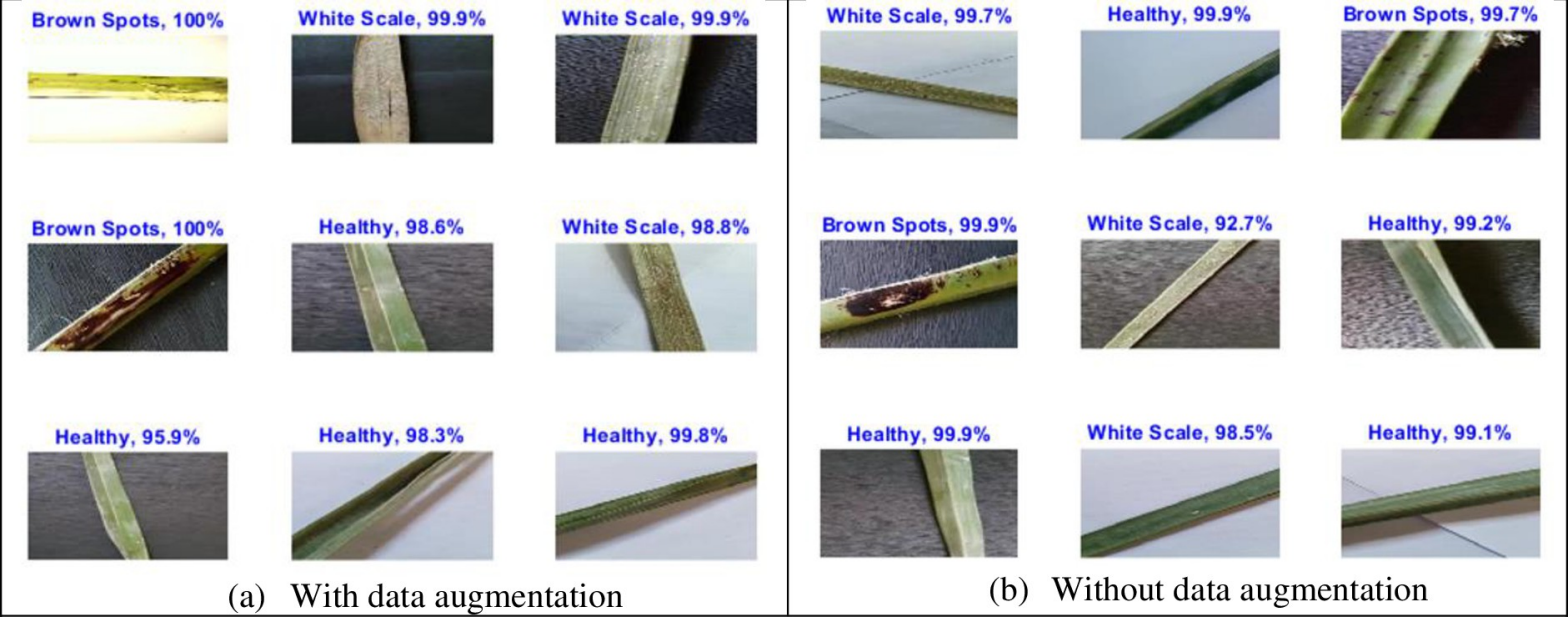
Palm-Leaves classification result using the proposed ResNet 1x1, 3x3 with and without data augmentation. (a) With data augmentation; (b) Without data augmentation.

**Fig 6:**
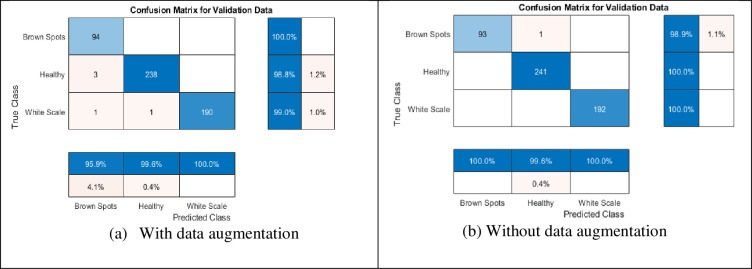
The confusion matrix of the classification accuracy using the proposed ResNet 1x1 and 3x3 with and without data augmentation. (a) With data augmentation; (b) Without data augmentation.

The training process is accomplished on the dataset with a number of epochs from 5 to 30 and a total number of iterations per epoch is 210 as shown in Fig 4. To verify the trained network, it is tested with a number of Palm-Leaves images from all the included categories. Fig 5 shows samples of tested images with their labels and the classification score for each category. Fig 6 displays the confusion matrix rows that correspond to the true class and the confusion matrix columns corresponding to the predicted class. The confusion matrix diagonal and off-diagonal cells are corresponding to the correct and incorrect classified observations, respectively. The network most commonly confuses healthy with brown spots classes and perfectly classifies the other classes. According to the results shown in the figures, the ResNet deep learning network decreased the validation loss gradually with stable learning (Overfitting). The choice of ResNet architecture in our work is based on the above-mentioned advantage. The proposed ResNet model gives higher validation accuracy of 99.05% at 25 epochs and lower validation accuracy of 95.26% at 10 epochs as shown in Table 5. On the other hand, it obviously from the figures that ResNet model achieved low validation accuracy with augmented dataset than the original one.

**Table 5:** The performance evaluation of the three proposed models against the models appeared in reference [20] and reference [21].

# of epochs	Metrics	Inception V4 [20]	ResNet 50 [20]	ResNet 152 [20]	Model in [21]	Vgg16 [21]	Proposed ResNet	Proposed MResNet	Proposed IncResNet
10 epochs	Training accuracy	99.89	99.95	**99.99**	NA	NA	97.10	98.72	99.14
	Training loss	0.0272	**0.0188**	0.2815	NA	NA	2.899	1.2833	0.8555
	Validation accuracy	99.38	**99.57**	97.98	NA	NA	95.26	99.05	97.15
	Validation loss	NA	NA	NA	NA	NA	4.74	**0.95**	2.85
	Overall Precision	NA	NA	NA	NA	NA	95.38	**99.02**	98.05
	Overall recall	NA	NA	NA	NA	NA	95.94	**99.20**	98.05
	F1 Score	NA	NA	NA	NA	NA	95.66	**99.11**	97.54
25 epochs	Training accuracy	NA	NA	NA	NA	NA	99.48	**99.52**	98.67
	Training loss	NA	NA	NA	NA	NA	0.5228	**0.4753**	1.3308
	Validation accuracy	NA	NA	NA	99.10	99.35	99.05	**99.62**	94.88
	Validation loss	NA	NA	NA	NA	NA	0.95	**0.38**	5.12
	Overall Precision	NA	NA	NA	99.23	99.23	98.50	**99.69**	96.64
	Overall recall	NA	NA	NA	98.54	99.08	99.24	**99.47**	96.64
	F1 Score	NA	NA	NA	99.18	99.36	98.87	**99.58**	94.29
30 epochs	Training accuracy	99.74	99.99	**100**	NA	NA	99.14	99.86	99.05
	Training loss	0.0102	6.238e-04	**2.4844e-04**	NA	NA	0.8555	0.1426	0.9506
	Validation accuracy	98.02	99.67	99.68	NA	NA	98.67	**100**	95.07
	Validation loss	0.0673	0.0159	0.0156	NA	NA	1.33	**0**	4.93
	Overall Precision	NA	NA	NA	NA	NA	98.12	**100**	92.97
	Overall recall	NA	NA	NA	NA	NA	98.99	**100**	92.97
	F1 Score	NA	NA	NA	NA	NA	98.56	**100**	94.47

### E) Proposed MResNet results

**Fig 7:**
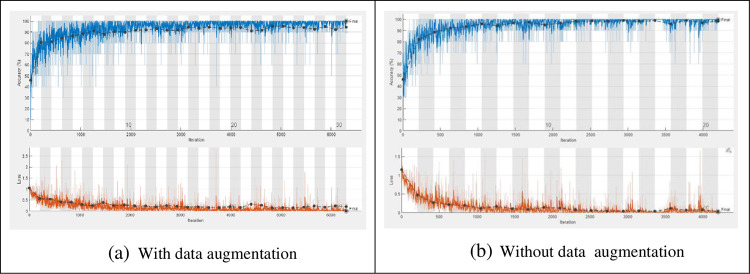
Training progress of the proposed MResNet 3x3, 5x5, with and without data augmentation. (a) With data augmentation; (b) Without data augmentation.

**Fig 8:**
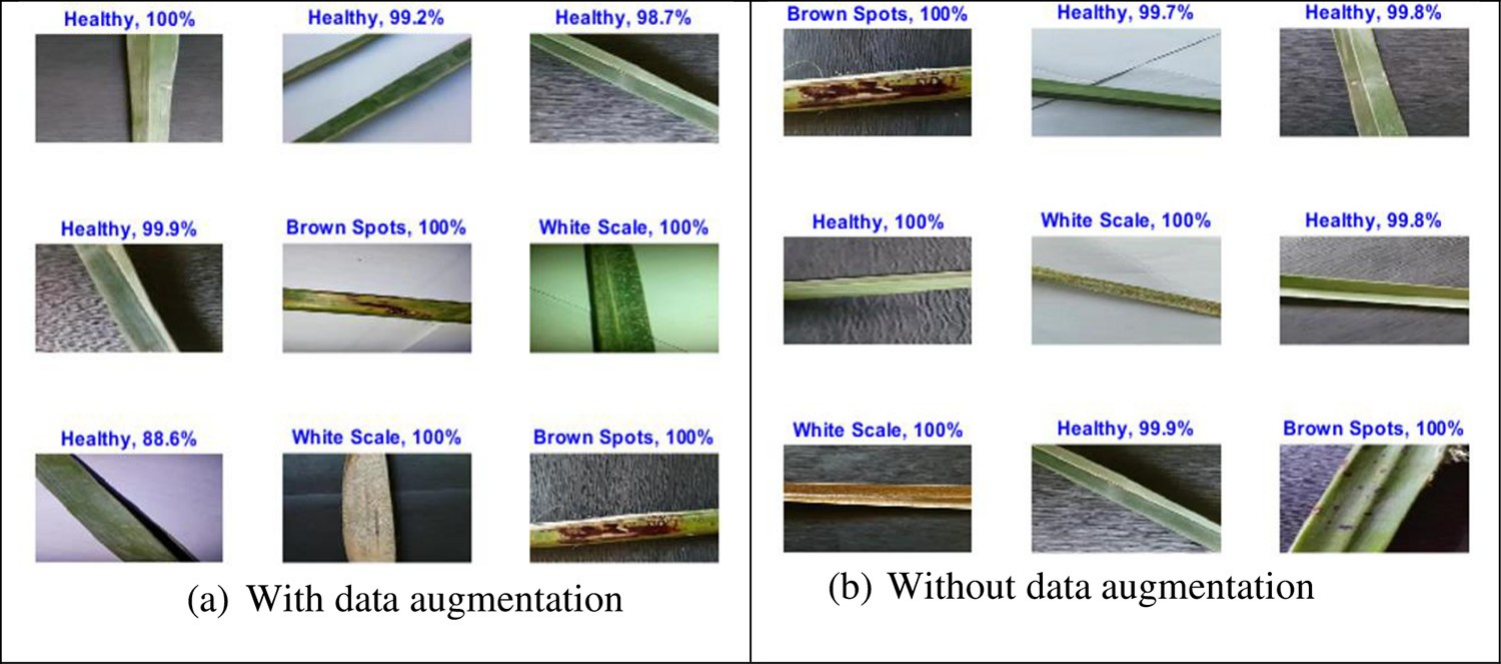
Palm-leaves classification result using the proposed MResNet 3x3, 5x5, with and without data augmentation. (a) With data augmentation; (b) Without data augmentation.

**Fig 9:**
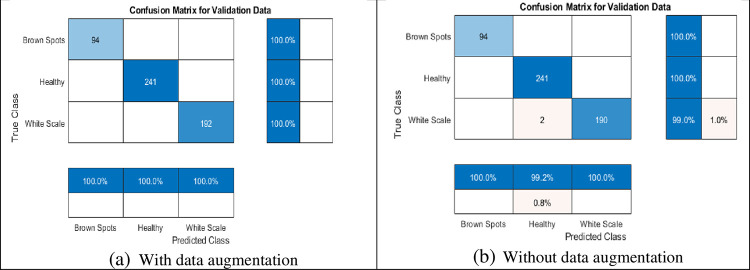
The confusion matrix of the classification accuracy using the proposed MResNet 3x3, 5x5, with and without data augmentation. (a) With data augmentation; (b) Without data augmentation.

Since the filter sizes and numbers in each deep learning layer play a necessary role in the accuracy of the network, the proposed MResNet model depends on filters of sizes 3x3 and 5x5. As known, choosing an appropriate filter size mainly affects the feature extraction process and this leads to more validation accuracy with minimal overfitting in the training progress. In this model, an appropriate neighborhood area is considered in the convolution process during the training to overcome the drawbacks of using the small neighborhood area by the first proposed ResNet model. As seen in Fig 7, the proposed modified model decreases the overfitting in the training progress when compared to the first proposed model. Fig 8 shows a few Palm-Leaves classification result using the proposed ResNet at different filter sizes 3x3 and 5x5. Fig 9 illustrates the confusion matrix of the proposed ResNet depending on sizes 3x3 and 5x5. It’s clear from the figure that the proposed network perfectly classifies all classes. Moreover, as shown in Table 5, the proposed MResNet model gives higher validation accuracy of 100% at 30 epochs and a slightly lower validation accuracy of 99.05% at 10 epochs. On the other hand, the proposed MResNet model was tested using original and augmented dataset and achieved accuracy of 99.62% and 100% respectively.

### F) IncResNet results

**Fig 10:**
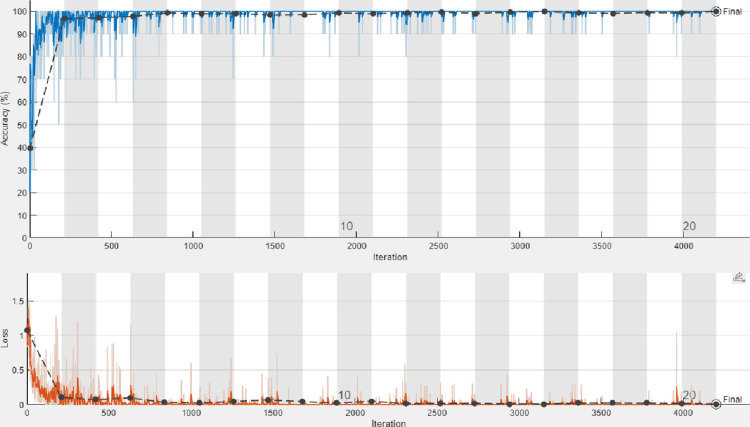
Training progress of the IncResNet.

**Fig 11:**
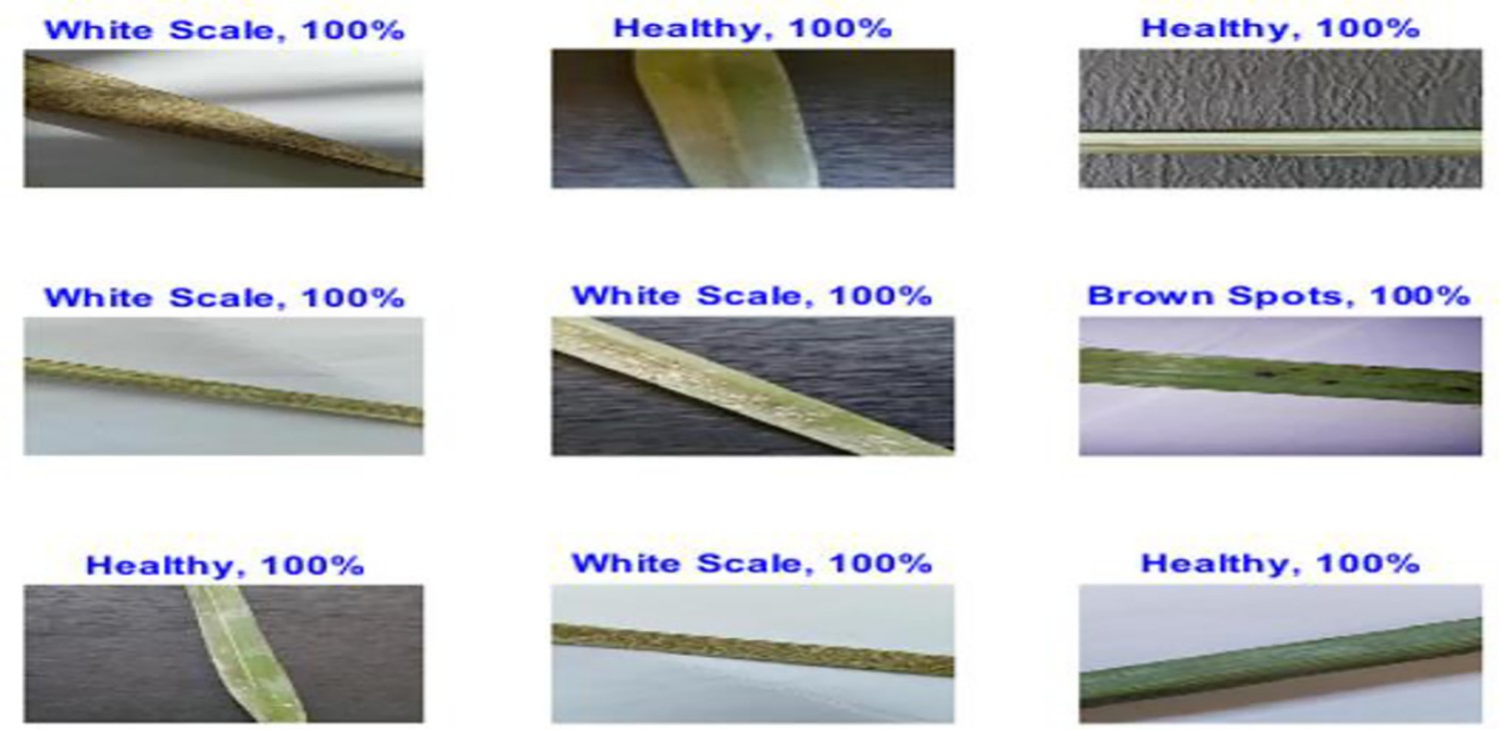
Palm-leaves classification result using the IncResNet.

**Fig 12:**
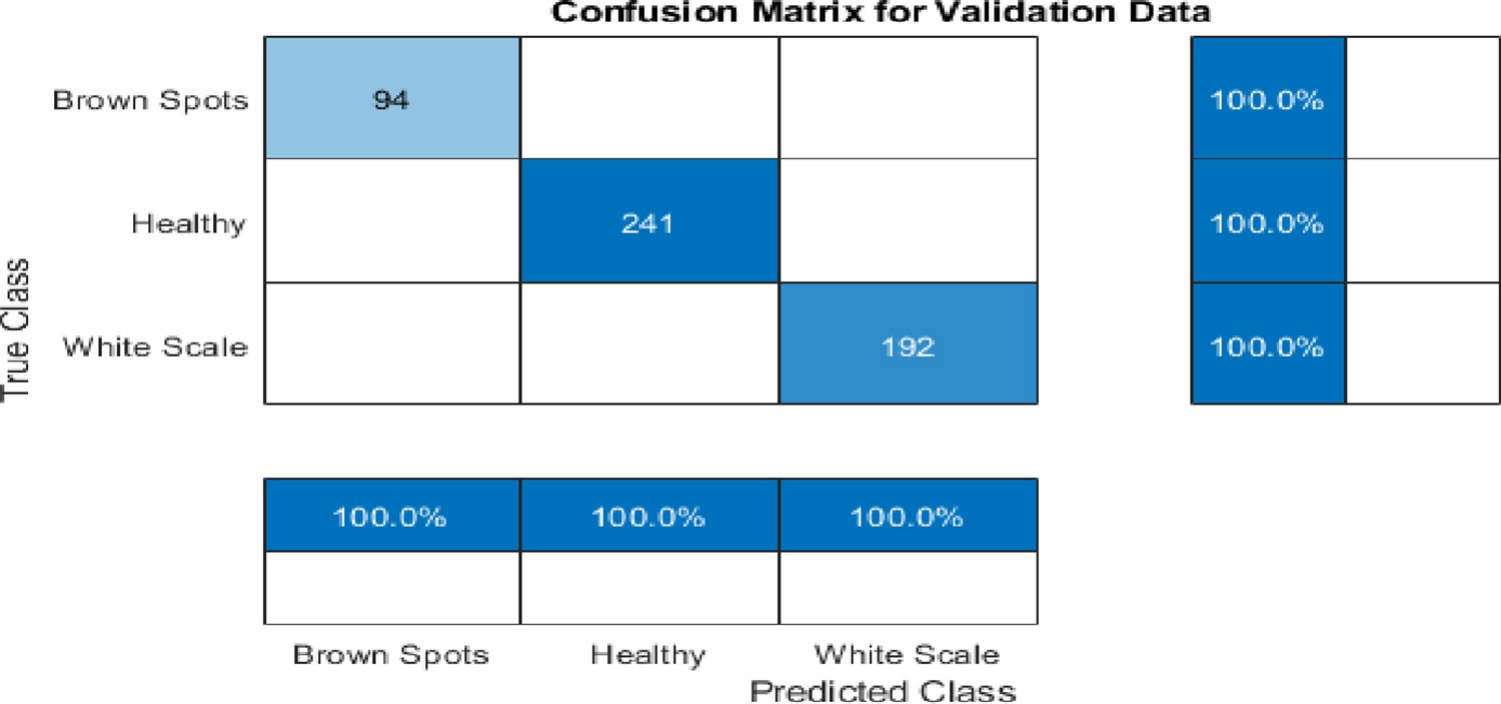
The confusion matrix of the classification accuracy using the IncResNet at 20 epochs.

In our work, the pre-trained IncResNet CNN model [24] is trained on an augmented palm leaf dataset used for image classification. The model has learned many feature representations for a variety of images. The image input of the network has a size of 299 by 299. Table 4 shows the hyperparameters employed during the training process. As shown in Table 5, the IncResNet model gives higher validation accuracy of 100% at 20 epochs and a lower validation accuracy of 95.07% at 30 epochs. Fig 10 shows the training process of the proposed IncResNet network. According to the results shown in the figure, IncResNet deep learning network decreased the validation loss gradually with stable learning. Fig 11 shows a few Palm-Leaves classification results using IncResNet. As shown in the figure, the network perfectly classifies healthy and unhealthy images. Fig 12 illustrated the confusion matrix of the proposed Inception IncResNet at 20 epochs. As shown in the figure, the proposed network works perfectly in all classes.

### G) Comparative analysis of the implemented models

**Fig 13:**
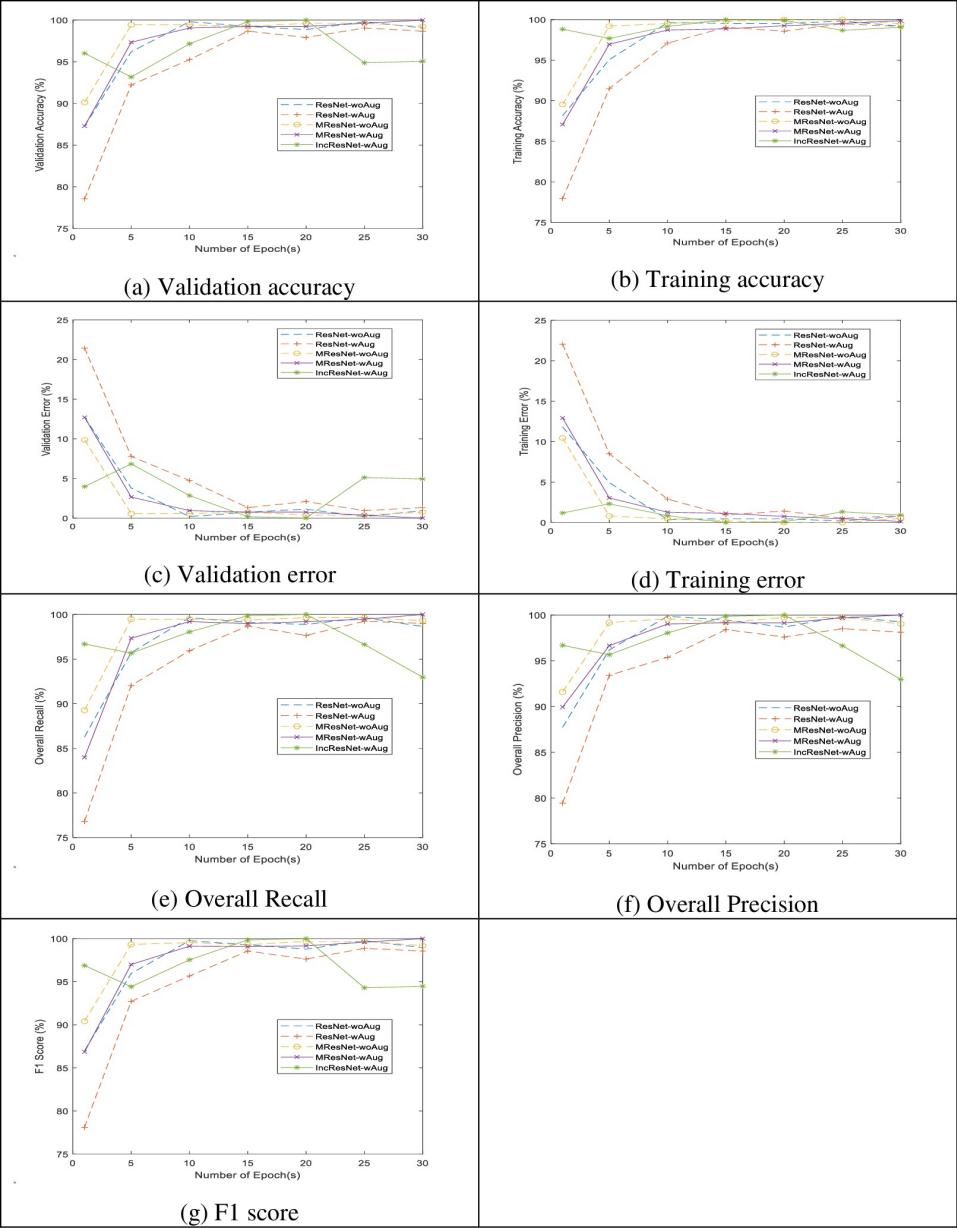
The performance evaluation of the three implemented models: ResNet, MResNet, and IncResNet. (a) Validation accuracy; (b) Training accuracy; (c) Validation error; (d) Training error; (e) Overall recall; (f) Overall precision; (g) F1 score.

The comparative analysis of the three implemented models at different epochs is declared in Fig 13. As shown from the figure, MResNet gave higher validation accuracy, overall recall, overall precision, and F1-score than ResNet and IncResNet at epochs 10, 15,25, and 30. On the other hand, IncResNet outperforms MResNet and ResNet at epochs 20 using all evaluation metrics. In addition, it is clear from the figure that MResNet gives high accuracy with the augmented dataset than when tested against the original dataset.

**Fig 14:**
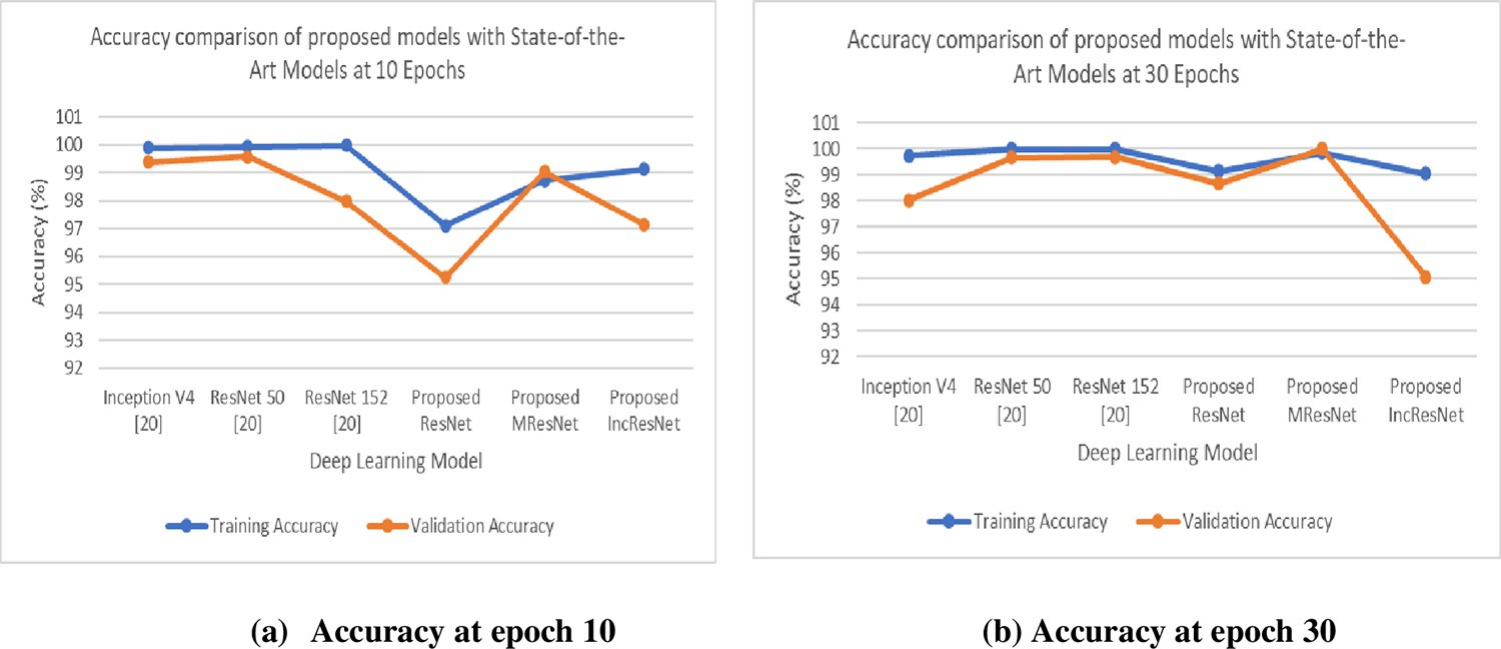
Training and validation accuracy of the proposed models at epochs 10 and 30 compared with models in [20]. (a) Accuracy at epoch 10; (b) Accuracy at epoch 30.

**Fig 15:**
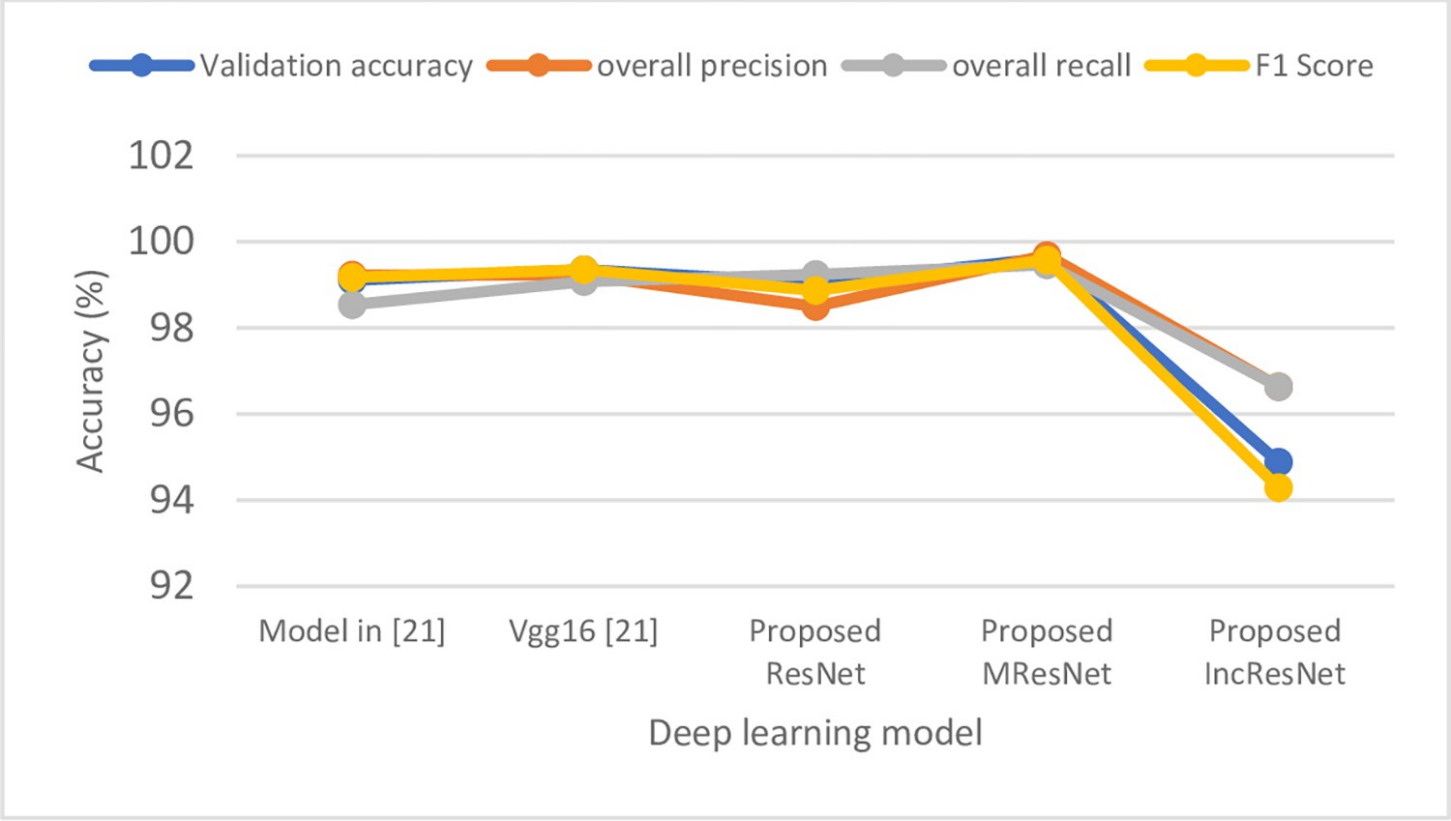
Validation accuracy, overall precision, overall recall, and F1 score of the proposed models compared with models in [21] at epoch 25.

To test the performance evaluation of the three implemented models against some state-of-the-art work, we chose two recent references related to palm leaf disease classification ([20] and [21]). The comparison was done according to the available tested metrics as well as the number of epochs chosen. As shown in Table 5, the three implemented models were tested using the seven metrics as well as three different numbers of epochs while [20] and [21] were tested using a few metrics and a single or a double number of epochs. It is clear from the table that the accuracy of the models proposed in [20] is slightly higher than our implemented models at 10 epochs while our proposed MResNet gave higher validation accuracy at epochs 30 as shown in Fig 14. On the other hand, our proposed MResNet gave higher validation accuracy, overall precision, overall recall, and F1 score than the models implemented in [21] at 25 epochs. Also, using the available evaluation metrics, the accuracy of the implemented models compared with the models in [21] at epoch 25 are declared in Fig 15. It was shown from the figure that the our proposed MResNet outperform the models implemented in [21] as well as the two proposed ResNet and IncResNet.

The overall results show that the proposed MResNet is better in classification than the other models in [20] and [21] as well as the proposed ResNet and Inception ResNet. Although IncResNet gave validation accuracy slightly lower than Inception ResNet V4 in [20] at 10 and 30 epochs, it gave 100% validation accuracy at 20 epochs as shown in Table 5. Moreover, the proposed MResNet model acquires the best validation accuracy of 100% at 30 epochs. On the other hand, the training accuracies of the models in [20] at epochs 10 and 30 are slightly higher than that of the three implemented models proposed in this study. Furthermore, the proposed MResNet model gave higher recall, precision, and F1 score than the models used in [21]. The reason for the better accuracy of MResNet model is that it takes into consideration the appropriate neighborhood area in the convolution process during the training to overcome the drawbacks of using the small neighborhood area by the proposed ResNet model. So, in this model, the higher number of iterations, the better validation accuracy is obtained.

## IV. Conclusion

One of the characteristics of palm disease is that the disease symptoms are usually appear as spotted on the leaves at its nursery stage. So, the main purpose is to develop a system that can detect and separate the infected palm trees earlier to prevent the reduction of palm crops. Deep learning models have become hot models to be used in detecting and classifying plant diseases using images. In this study, two approaches of deep learning have been implemented for Palm leaf disease classification, namely Residual Network (ResNet) and transfer learning of Inception ResNet. The reason to choose the model for palm tree diseases is to take the advantage of ResNet and transfer learning ResNet to train hundreds of layers effectively. Among the implemented deep learning models to classify palm leaf disease, the obtained results show that the proposed MResNet gave higher validation accuracy and reached 100% at different epochs. In addition, the superiority of the MResNet model was proven when compared to some of the recent state-of-the-art deep learning models using some of the well-known metrics. On the other hand, the biggest challenges with using ResNet models for classifying such challenging palm leaves dataset is the model computational density. Because of their heavy computation, ResNets models are mainly run on GPUs. In future studies, the DL model architecture based on one of the appropriate metaheuristic algorithms for the extracting smallest feature subset will decrease the computation cost as well as increase the classification accuracy and can be tested to classify a variety of plant diseases.

## V. Data availability

The data that support the findings of this study are openly available in [Date Palm data | Kaggle] at https://www.kaggle.com/datasets/hadjerhamaidi/date-palm-data.

## Supporting information

S1 Data Date Palm data. (ZIP)
