# Supplementary figures and images for: Palm tree disease detection and classification using residual network and transfer learning of inception ResNet (part 2 of 2)
Source: PLoS One. 2023 Mar 2;18(3):e0282250. doi: 10.1371/journal.pone.0282250 (PMC9980777; doi:10.1371/journal.pone.0282250)

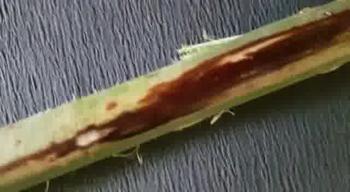

Supplement: S1 Data — (ZIP) [file pone.0282250.s001.zip › DatePalmData/Brown Spots/brownspots-19.jpg]

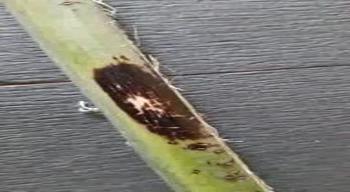

Supplement: S1 Data — (ZIP) [file pone.0282250.s001.zip › DatePalmData/Brown Spots/brownspots-190.jpg]

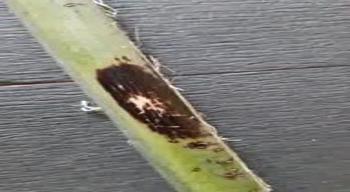

Supplement: S1 Data — (ZIP) [file pone.0282250.s001.zip › DatePalmData/Brown Spots/brownspots-191.jpg]

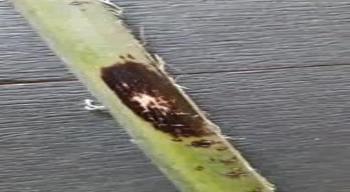

Supplement: S1 Data — (ZIP) [file pone.0282250.s001.zip › DatePalmData/Brown Spots/brownspots-192.jpg]

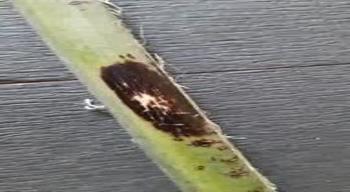

Supplement: S1 Data — (ZIP) [file pone.0282250.s001.zip › DatePalmData/Brown Spots/brownspots-193.jpg]

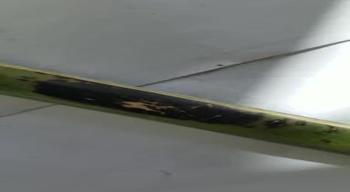

Supplement: S1 Data — (ZIP) [file pone.0282250.s001.zip › DatePalmData/Brown Spots/brownspots-194.jpg]

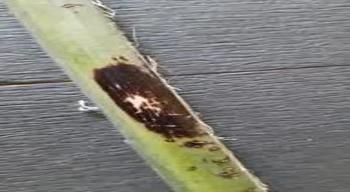

Supplement: S1 Data — (ZIP) [file pone.0282250.s001.zip › DatePalmData/Brown Spots/brownspots-195.jpg]

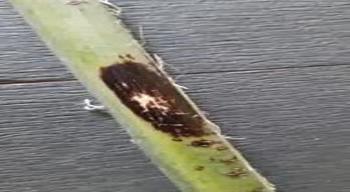

Supplement: S1 Data — (ZIP) [file pone.0282250.s001.zip › DatePalmData/Brown Spots/brownspots-196.jpg]

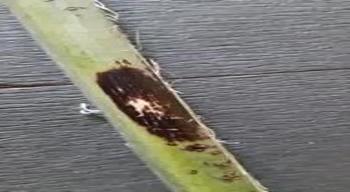

Supplement: S1 Data — (ZIP) [file pone.0282250.s001.zip › DatePalmData/Brown Spots/brownspots-197.jpg]

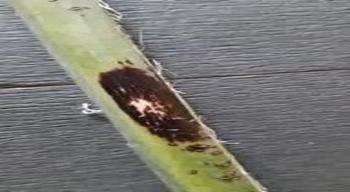

Supplement: S1 Data — (ZIP) [file pone.0282250.s001.zip › DatePalmData/Brown Spots/brownspots-198.jpg]

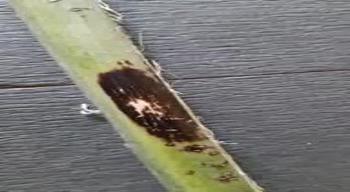

Supplement: S1 Data — (ZIP) [file pone.0282250.s001.zip › DatePalmData/Brown Spots/brownspots-199.jpg]

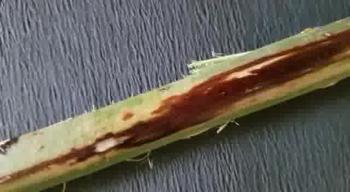

Supplement: S1 Data — (ZIP) [file pone.0282250.s001.zip › DatePalmData/Brown Spots/brownspots-2.jpg]

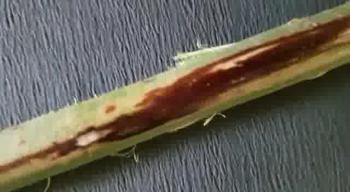

Supplement: S1 Data — (ZIP) [file pone.0282250.s001.zip › DatePalmData/Brown Spots/brownspots-20.jpg]

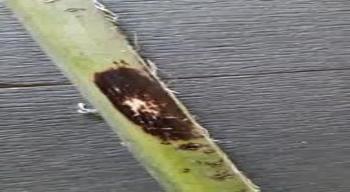

Supplement: S1 Data — (ZIP) [file pone.0282250.s001.zip › DatePalmData/Brown Spots/brownspots-200.jpg]

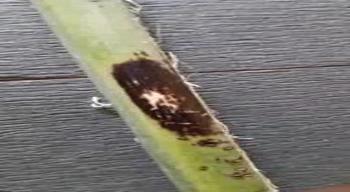

Supplement: S1 Data — (ZIP) [file pone.0282250.s001.zip › DatePalmData/Brown Spots/brownspots-201.jpg]

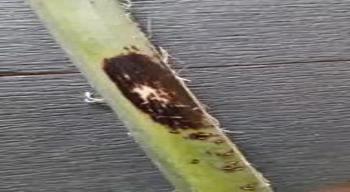

Supplement: S1 Data — (ZIP) [file pone.0282250.s001.zip › DatePalmData/Brown Spots/brownspots-202.jpg]

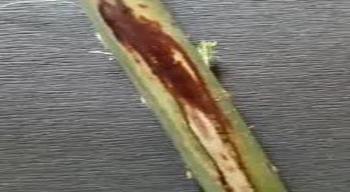

Supplement: S1 Data — (ZIP) [file pone.0282250.s001.zip › DatePalmData/Brown Spots/brownspots-203.jpg]

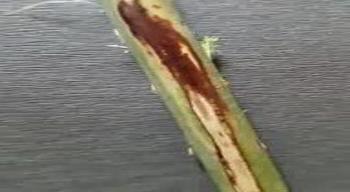

Supplement: S1 Data — (ZIP) [file pone.0282250.s001.zip › DatePalmData/Brown Spots/brownspots-204.jpg]

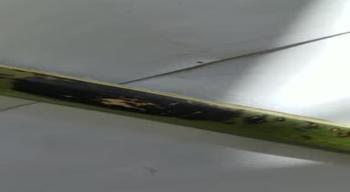

Supplement: S1 Data — (ZIP) [file pone.0282250.s001.zip › DatePalmData/Brown Spots/brownspots-205.jpg]

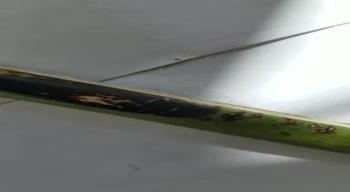

Supplement: S1 Data — (ZIP) [file pone.0282250.s001.zip › DatePalmData/Brown Spots/brownspots-206.jpg]

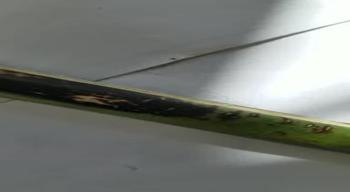

Supplement: S1 Data — (ZIP) [file pone.0282250.s001.zip › DatePalmData/Brown Spots/brownspots-207.jpg]

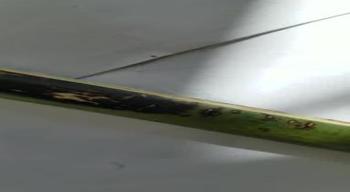

Supplement: S1 Data — (ZIP) [file pone.0282250.s001.zip › DatePalmData/Brown Spots/brownspots-208.jpg]

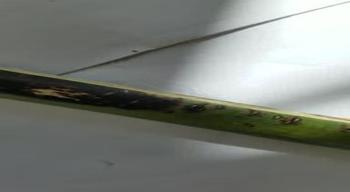

Supplement: S1 Data — (ZIP) [file pone.0282250.s001.zip › DatePalmData/Brown Spots/brownspots-209.jpg]

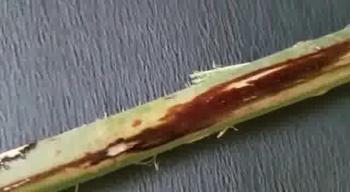

Supplement: S1 Data — (ZIP) [file pone.0282250.s001.zip › DatePalmData/Brown Spots/brownspots-21.jpg]

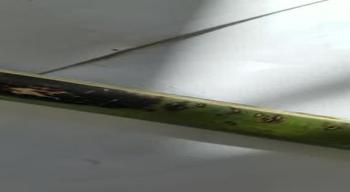

Supplement: S1 Data — (ZIP) [file pone.0282250.s001.zip › DatePalmData/Brown Spots/brownspots-210.jpg]

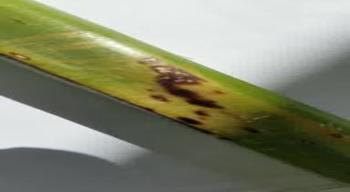

Supplement: S1 Data — (ZIP) [file pone.0282250.s001.zip › DatePalmData/Brown Spots/brownspots-211.jpg]

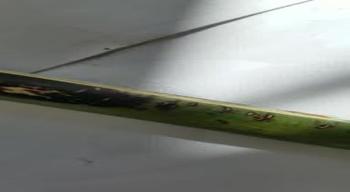

Supplement: S1 Data — (ZIP) [file pone.0282250.s001.zip › DatePalmData/Brown Spots/brownspots-212.jpg]

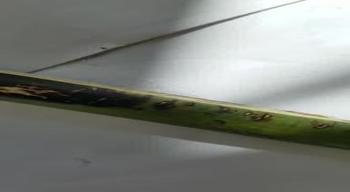

Supplement: S1 Data — (ZIP) [file pone.0282250.s001.zip › DatePalmData/Brown Spots/brownspots-213.jpg]

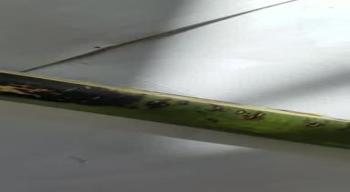

Supplement: S1 Data — (ZIP) [file pone.0282250.s001.zip › DatePalmData/Brown Spots/brownspots-214.jpg]

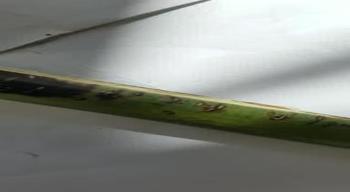

Supplement: S1 Data — (ZIP) [file pone.0282250.s001.zip › DatePalmData/Brown Spots/brownspots-215.jpg]

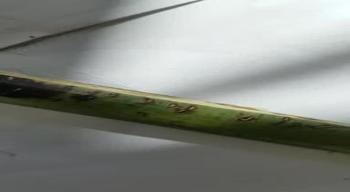

Supplement: S1 Data — (ZIP) [file pone.0282250.s001.zip › DatePalmData/Brown Spots/brownspots-216.jpg]

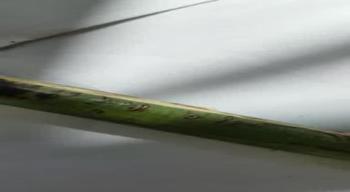

Supplement: S1 Data — (ZIP) [file pone.0282250.s001.zip › DatePalmData/Brown Spots/brownspots-217.jpg]

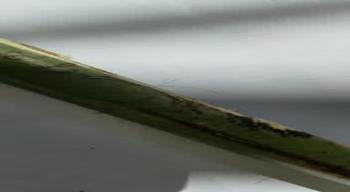

Supplement: S1 Data — (ZIP) [file pone.0282250.s001.zip › DatePalmData/Brown Spots/brownspots-218.jpg]

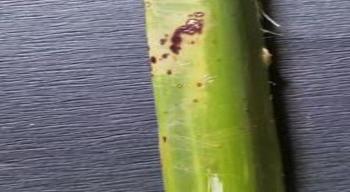

Supplement: S1 Data — (ZIP) [file pone.0282250.s001.zip › DatePalmData/Brown Spots/brownspots-219.jpg]

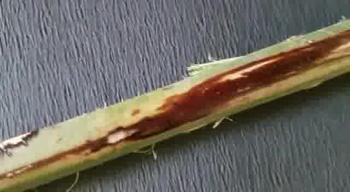

Supplement: S1 Data — (ZIP) [file pone.0282250.s001.zip › DatePalmData/Brown Spots/brownspots-22.jpg]

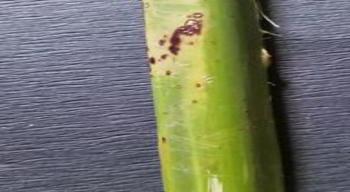

Supplement: S1 Data — (ZIP) [file pone.0282250.s001.zip › DatePalmData/Brown Spots/brownspots-220.jpg]

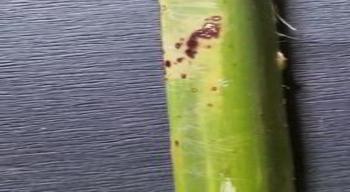

Supplement: S1 Data — (ZIP) [file pone.0282250.s001.zip › DatePalmData/Brown Spots/brownspots-221.jpg]

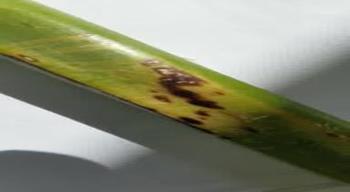

Supplement: S1 Data — (ZIP) [file pone.0282250.s001.zip › DatePalmData/Brown Spots/brownspots-222.jpg]

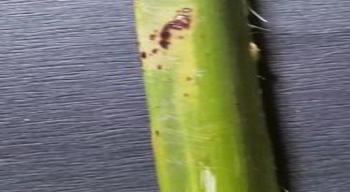

Supplement: S1 Data — (ZIP) [file pone.0282250.s001.zip › DatePalmData/Brown Spots/brownspots-223.jpg]

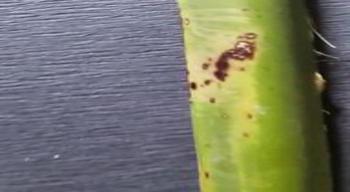

Supplement: S1 Data — (ZIP) [file pone.0282250.s001.zip › DatePalmData/Brown Spots/brownspots-224.jpg]

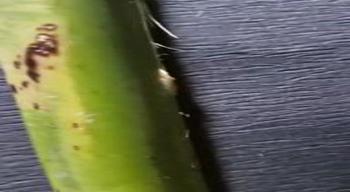

Supplement: S1 Data — (ZIP) [file pone.0282250.s001.zip › DatePalmData/Brown Spots/brownspots-225.jpg]

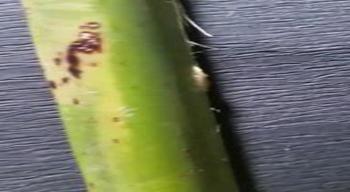

Supplement: S1 Data — (ZIP) [file pone.0282250.s001.zip › DatePalmData/Brown Spots/brownspots-226.jpg]

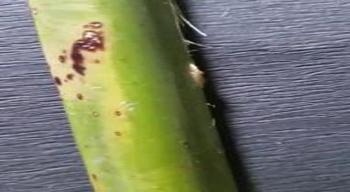

Supplement: S1 Data — (ZIP) [file pone.0282250.s001.zip › DatePalmData/Brown Spots/brownspots-227.jpg]

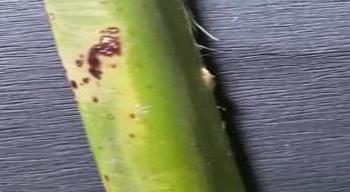

Supplement: S1 Data — (ZIP) [file pone.0282250.s001.zip › DatePalmData/Brown Spots/brownspots-228.jpg]

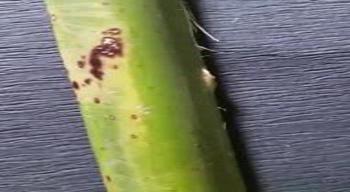

Supplement: S1 Data — (ZIP) [file pone.0282250.s001.zip › DatePalmData/Brown Spots/brownspots-229.jpg]

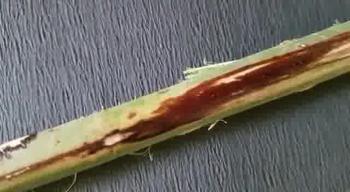

Supplement: S1 Data — (ZIP) [file pone.0282250.s001.zip › DatePalmData/Brown Spots/brownspots-23.jpg]

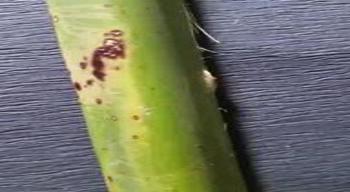

Supplement: S1 Data — (ZIP) [file pone.0282250.s001.zip › DatePalmData/Brown Spots/brownspots-230.jpg]

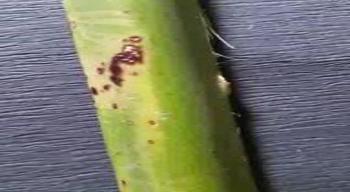

Supplement: S1 Data — (ZIP) [file pone.0282250.s001.zip › DatePalmData/Brown Spots/brownspots-231.jpg]

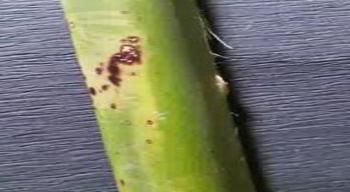

Supplement: S1 Data — (ZIP) [file pone.0282250.s001.zip › DatePalmData/Brown Spots/brownspots-232.jpg]

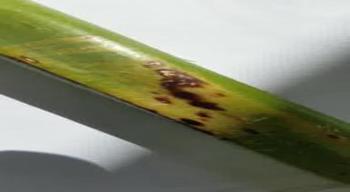

Supplement: S1 Data — (ZIP) [file pone.0282250.s001.zip › DatePalmData/Brown Spots/brownspots-233.jpg]

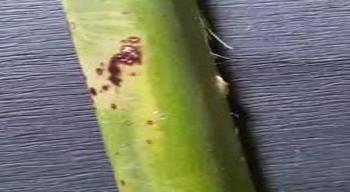

Supplement: S1 Data — (ZIP) [file pone.0282250.s001.zip › DatePalmData/Brown Spots/brownspots-234.jpg]

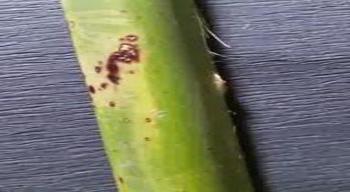

Supplement: S1 Data — (ZIP) [file pone.0282250.s001.zip › DatePalmData/Brown Spots/brownspots-235.jpg]

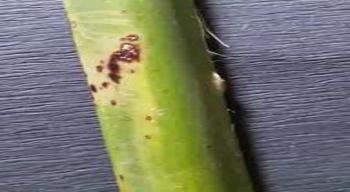

Supplement: S1 Data — (ZIP) [file pone.0282250.s001.zip › DatePalmData/Brown Spots/brownspots-236.jpg]

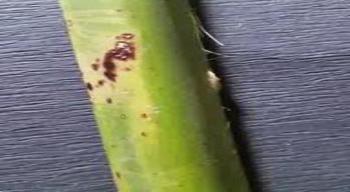

Supplement: S1 Data — (ZIP) [file pone.0282250.s001.zip › DatePalmData/Brown Spots/brownspots-237.jpg]

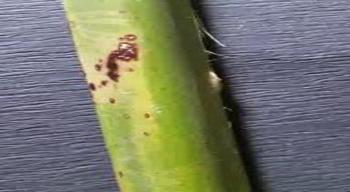

Supplement: S1 Data — (ZIP) [file pone.0282250.s001.zip › DatePalmData/Brown Spots/brownspots-238.jpg]

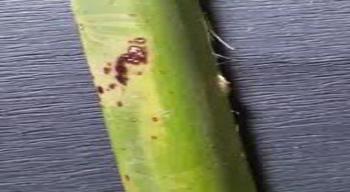

Supplement: S1 Data — (ZIP) [file pone.0282250.s001.zip › DatePalmData/Brown Spots/brownspots-239.jpg]

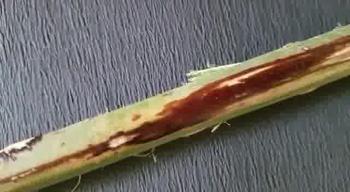

Supplement: S1 Data — (ZIP) [file pone.0282250.s001.zip › DatePalmData/Brown Spots/brownspots-24.jpg]

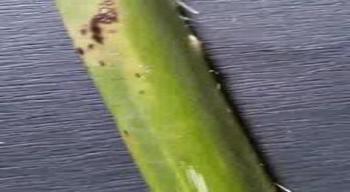

Supplement: S1 Data — (ZIP) [file pone.0282250.s001.zip › DatePalmData/Brown Spots/brownspots-240.jpg]

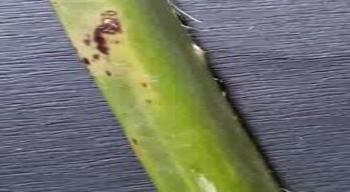

Supplement: S1 Data — (ZIP) [file pone.0282250.s001.zip › DatePalmData/Brown Spots/brownspots-241.jpg]

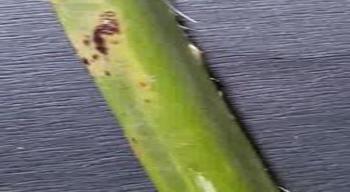

Supplement: S1 Data — (ZIP) [file pone.0282250.s001.zip › DatePalmData/Brown Spots/brownspots-242.jpg]

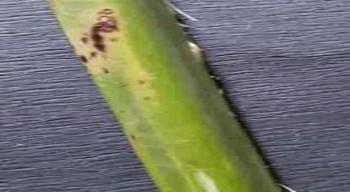

Supplement: S1 Data — (ZIP) [file pone.0282250.s001.zip › DatePalmData/Brown Spots/brownspots-243.jpg]

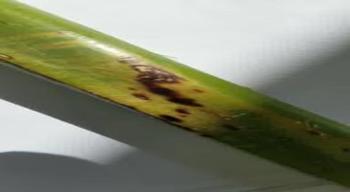

Supplement: S1 Data — (ZIP) [file pone.0282250.s001.zip › DatePalmData/Brown Spots/brownspots-244.jpg]

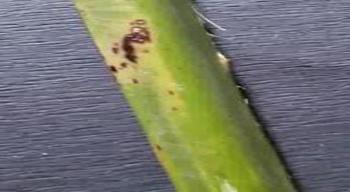

Supplement: S1 Data — (ZIP) [file pone.0282250.s001.zip › DatePalmData/Brown Spots/brownspots-245.jpg]

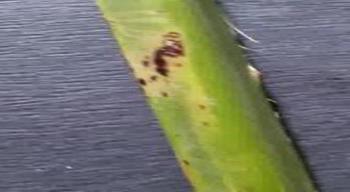

Supplement: S1 Data — (ZIP) [file pone.0282250.s001.zip › DatePalmData/Brown Spots/brownspots-246.jpg]

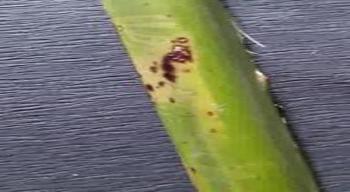

Supplement: S1 Data — (ZIP) [file pone.0282250.s001.zip › DatePalmData/Brown Spots/brownspots-247.jpg]

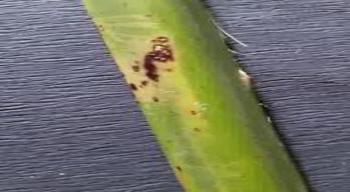

Supplement: S1 Data — (ZIP) [file pone.0282250.s001.zip › DatePalmData/Brown Spots/brownspots-248.jpg]

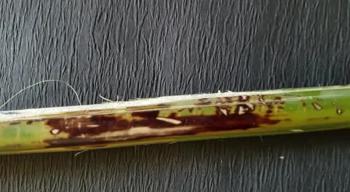

Supplement: S1 Data — (ZIP) [file pone.0282250.s001.zip › DatePalmData/Brown Spots/brownspots-249.jpg]

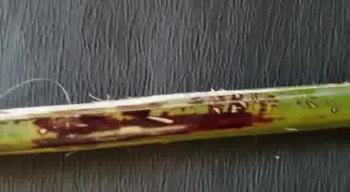

Supplement: S1 Data — (ZIP) [file pone.0282250.s001.zip › DatePalmData/Brown Spots/brownspots-25.jpg]

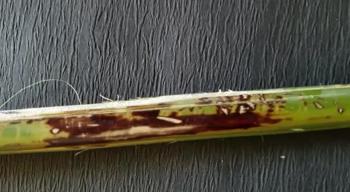

Supplement: S1 Data — (ZIP) [file pone.0282250.s001.zip › DatePalmData/Brown Spots/brownspots-250.jpg]

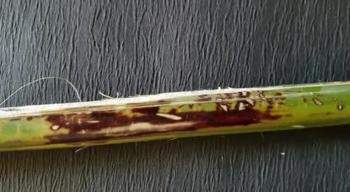

Supplement: S1 Data — (ZIP) [file pone.0282250.s001.zip › DatePalmData/Brown Spots/brownspots-251.jpg]

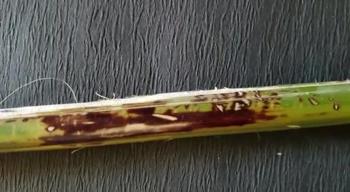

Supplement: S1 Data — (ZIP) [file pone.0282250.s001.zip › DatePalmData/Brown Spots/brownspots-252.jpg]

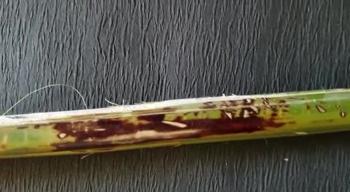

Supplement: S1 Data — (ZIP) [file pone.0282250.s001.zip › DatePalmData/Brown Spots/brownspots-253.jpg]

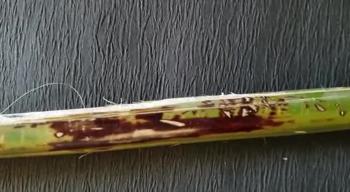

Supplement: S1 Data — (ZIP) [file pone.0282250.s001.zip › DatePalmData/Brown Spots/brownspots-254.jpg]

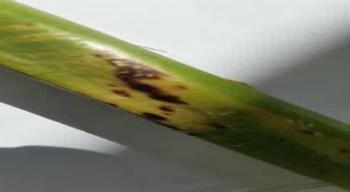

Supplement: S1 Data — (ZIP) [file pone.0282250.s001.zip › DatePalmData/Brown Spots/brownspots-255.jpg]

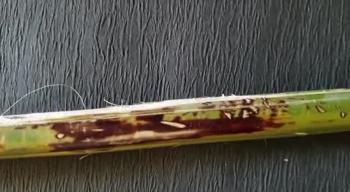

Supplement: S1 Data — (ZIP) [file pone.0282250.s001.zip › DatePalmData/Brown Spots/brownspots-256.jpg]

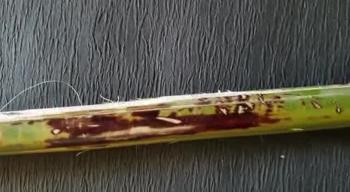

Supplement: S1 Data — (ZIP) [file pone.0282250.s001.zip › DatePalmData/Brown Spots/brownspots-257.jpg]

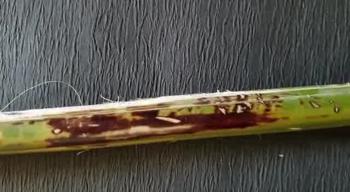

Supplement: S1 Data — (ZIP) [file pone.0282250.s001.zip › DatePalmData/Brown Spots/brownspots-258.jpg]

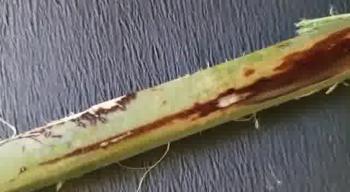

Supplement: S1 Data — (ZIP) [file pone.0282250.s001.zip › DatePalmData/Brown Spots/brownspots-259.jpg]

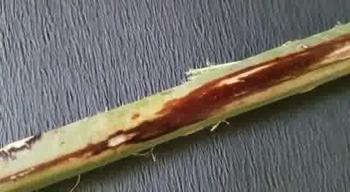

Supplement: S1 Data — (ZIP) [file pone.0282250.s001.zip › DatePalmData/Brown Spots/brownspots-26.jpg]

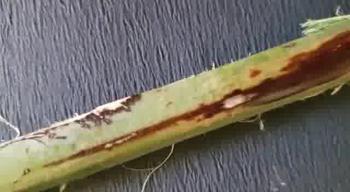

Supplement: S1 Data — (ZIP) [file pone.0282250.s001.zip › DatePalmData/Brown Spots/brownspots-260.jpg]

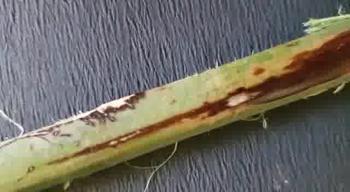

Supplement: S1 Data — (ZIP) [file pone.0282250.s001.zip › DatePalmData/Brown Spots/brownspots-261.jpg]

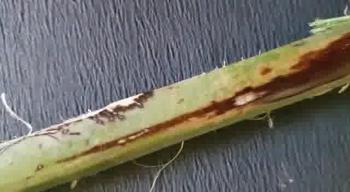

Supplement: S1 Data — (ZIP) [file pone.0282250.s001.zip › DatePalmData/Brown Spots/brownspots-262.jpg]

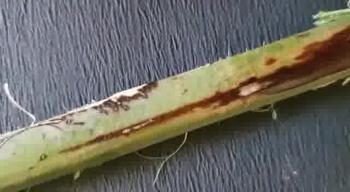

Supplement: S1 Data — (ZIP) [file pone.0282250.s001.zip › DatePalmData/Brown Spots/brownspots-263.jpg]

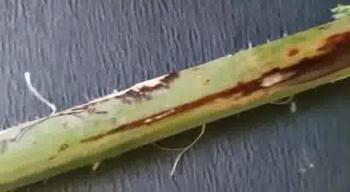

Supplement: S1 Data — (ZIP) [file pone.0282250.s001.zip › DatePalmData/Brown Spots/brownspots-264.jpg]

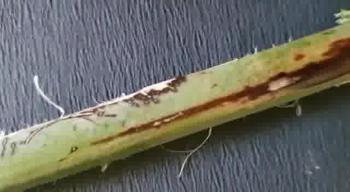

Supplement: S1 Data — (ZIP) [file pone.0282250.s001.zip › DatePalmData/Brown Spots/brownspots-265.jpg]

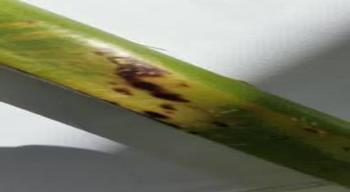

Supplement: S1 Data — (ZIP) [file pone.0282250.s001.zip › DatePalmData/Brown Spots/brownspots-266.jpg]

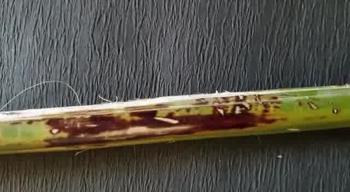

Supplement: S1 Data — (ZIP) [file pone.0282250.s001.zip › DatePalmData/Brown Spots/brownspots-267.jpg]

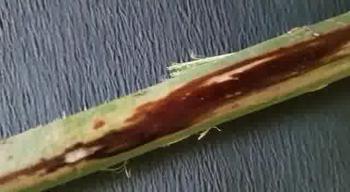

Supplement: S1 Data — (ZIP) [file pone.0282250.s001.zip › DatePalmData/Brown Spots/brownspots-268.jpg]

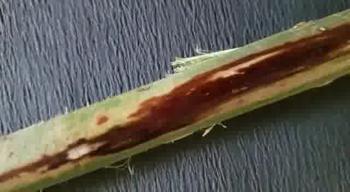

Supplement: S1 Data — (ZIP) [file pone.0282250.s001.zip › DatePalmData/Brown Spots/brownspots-269.jpg]

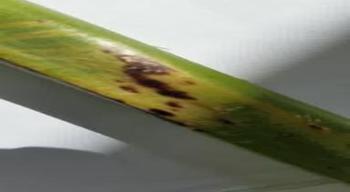

Supplement: S1 Data — (ZIP) [file pone.0282250.s001.zip › DatePalmData/Brown Spots/brownspots-27.jpg]

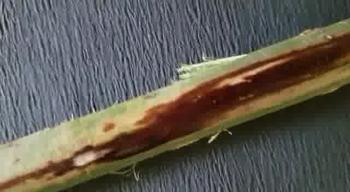

Supplement: S1 Data — (ZIP) [file pone.0282250.s001.zip › DatePalmData/Brown Spots/brownspots-270.jpg]

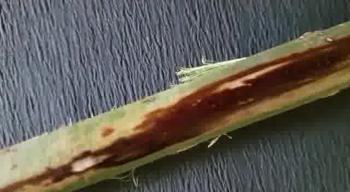

Supplement: S1 Data — (ZIP) [file pone.0282250.s001.zip › DatePalmData/Brown Spots/brownspots-271.jpg]

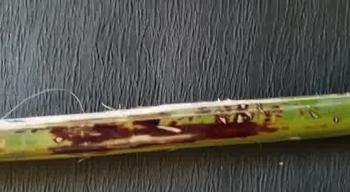

Supplement: S1 Data — (ZIP) [file pone.0282250.s001.zip › DatePalmData/Brown Spots/brownspots-272.jpg]

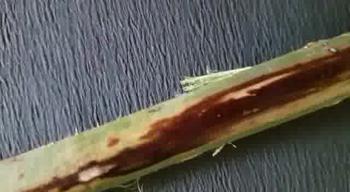

Supplement: S1 Data — (ZIP) [file pone.0282250.s001.zip › DatePalmData/Brown Spots/brownspots-273.jpg]

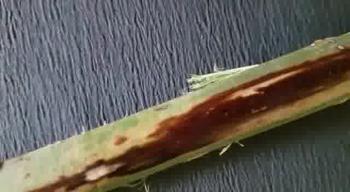

Supplement: S1 Data — (ZIP) [file pone.0282250.s001.zip › DatePalmData/Brown Spots/brownspots-274.jpg]

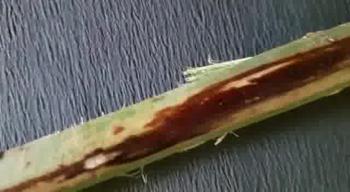

Supplement: S1 Data — (ZIP) [file pone.0282250.s001.zip › DatePalmData/Brown Spots/brownspots-275.jpg]

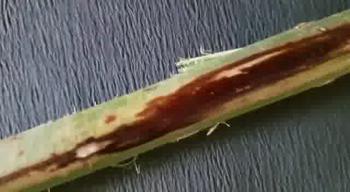

Supplement: S1 Data — (ZIP) [file pone.0282250.s001.zip › DatePalmData/Brown Spots/brownspots-276.jpg]

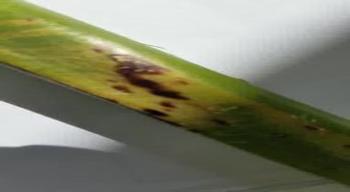

Supplement: S1 Data — (ZIP) [file pone.0282250.s001.zip › DatePalmData/Brown Spots/brownspots-277.jpg]

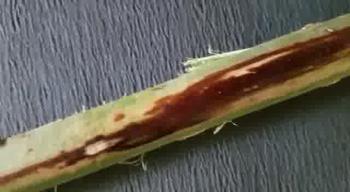

Supplement: S1 Data — (ZIP) [file pone.0282250.s001.zip › DatePalmData/Brown Spots/brownspots-278.jpg]

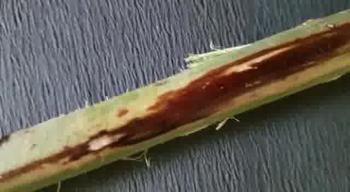

Supplement: S1 Data — (ZIP) [file pone.0282250.s001.zip › DatePalmData/Brown Spots/brownspots-279.jpg]
