# Supplementary figures and images for: Palm tree disease detection and classification using residual network and transfer learning of inception ResNet (part 1 of 2)
Source: PLoS One. 2023 Mar 2;18(3):e0282250. doi: 10.1371/journal.pone.0282250 (PMC9980777; doi:10.1371/journal.pone.0282250)

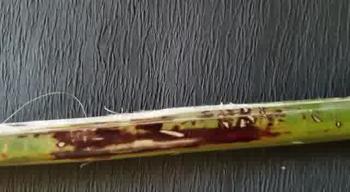

Supplement: S1 Data — (ZIP) [file pone.0282250.s001.zip › DatePalmData/Brown Spots/brownspots-1.jpg]

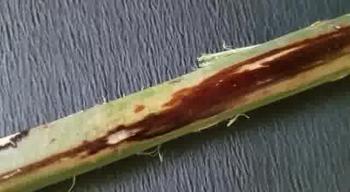

Supplement: S1 Data — (ZIP) [file pone.0282250.s001.zip › DatePalmData/Brown Spots/brownspots-10.jpg]

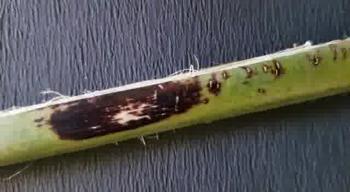

Supplement: S1 Data — (ZIP) [file pone.0282250.s001.zip › DatePalmData/Brown Spots/brownspots-100.jpg]

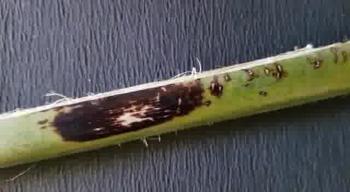

Supplement: S1 Data — (ZIP) [file pone.0282250.s001.zip › DatePalmData/Brown Spots/brownspots-101.jpg]

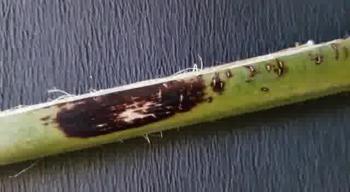

Supplement: S1 Data — (ZIP) [file pone.0282250.s001.zip › DatePalmData/Brown Spots/brownspots-102.jpg]

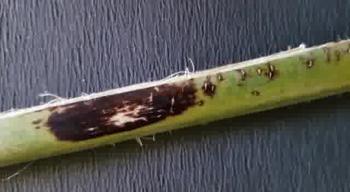

Supplement: S1 Data — (ZIP) [file pone.0282250.s001.zip › DatePalmData/Brown Spots/brownspots-103.jpg]

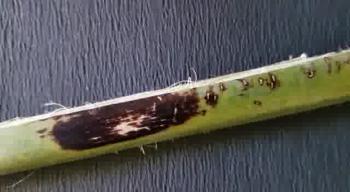

Supplement: S1 Data — (ZIP) [file pone.0282250.s001.zip › DatePalmData/Brown Spots/brownspots-104.jpg]

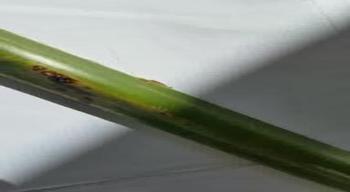

Supplement: S1 Data — (ZIP) [file pone.0282250.s001.zip › DatePalmData/Brown Spots/brownspots-105.jpg]

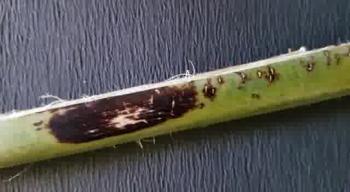

Supplement: S1 Data — (ZIP) [file pone.0282250.s001.zip › DatePalmData/Brown Spots/brownspots-106.jpg]

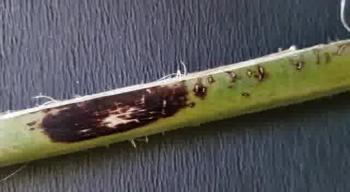

Supplement: S1 Data — (ZIP) [file pone.0282250.s001.zip › DatePalmData/Brown Spots/brownspots-107.jpg]

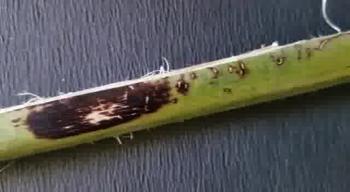

Supplement: S1 Data — (ZIP) [file pone.0282250.s001.zip › DatePalmData/Brown Spots/brownspots-108.jpg]

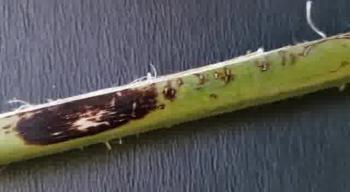

Supplement: S1 Data — (ZIP) [file pone.0282250.s001.zip › DatePalmData/Brown Spots/brownspots-109.jpg]

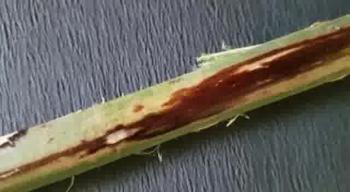

Supplement: S1 Data — (ZIP) [file pone.0282250.s001.zip › DatePalmData/Brown Spots/brownspots-11.jpg]

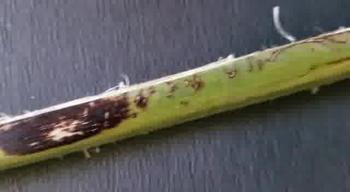

Supplement: S1 Data — (ZIP) [file pone.0282250.s001.zip › DatePalmData/Brown Spots/brownspots-110.jpg]

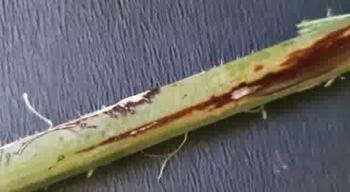

Supplement: S1 Data — (ZIP) [file pone.0282250.s001.zip › DatePalmData/Brown Spots/brownspots-111.jpg]

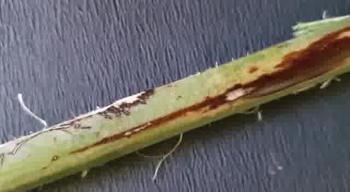

Supplement: S1 Data — (ZIP) [file pone.0282250.s001.zip › DatePalmData/Brown Spots/brownspots-112.jpg]

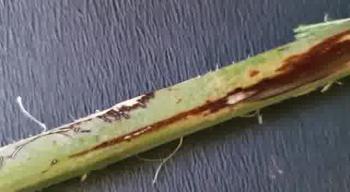

Supplement: S1 Data — (ZIP) [file pone.0282250.s001.zip › DatePalmData/Brown Spots/brownspots-113.jpg]

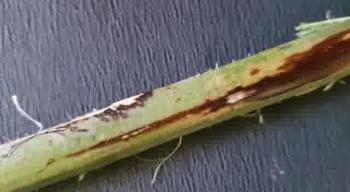

Supplement: S1 Data — (ZIP) [file pone.0282250.s001.zip › DatePalmData/Brown Spots/brownspots-114.jpg]

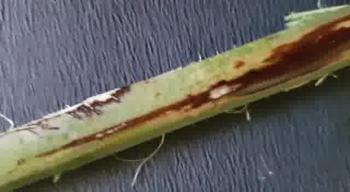

Supplement: S1 Data — (ZIP) [file pone.0282250.s001.zip › DatePalmData/Brown Spots/brownspots-115.jpg]

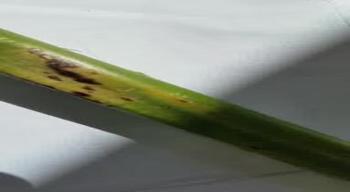

Supplement: S1 Data — (ZIP) [file pone.0282250.s001.zip › DatePalmData/Brown Spots/brownspots-116.jpg]

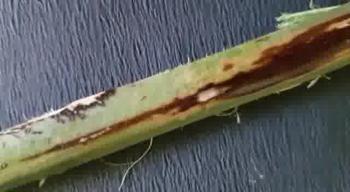

Supplement: S1 Data — (ZIP) [file pone.0282250.s001.zip › DatePalmData/Brown Spots/brownspots-117.jpg]

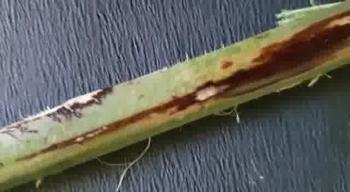

Supplement: S1 Data — (ZIP) [file pone.0282250.s001.zip › DatePalmData/Brown Spots/brownspots-118.jpg]

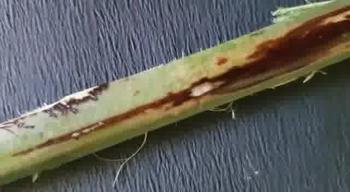

Supplement: S1 Data — (ZIP) [file pone.0282250.s001.zip › DatePalmData/Brown Spots/brownspots-119.jpg]

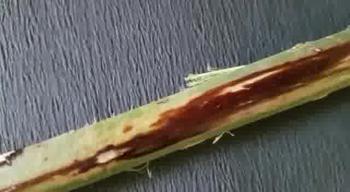

Supplement: S1 Data — (ZIP) [file pone.0282250.s001.zip › DatePalmData/Brown Spots/brownspots-12.jpg]

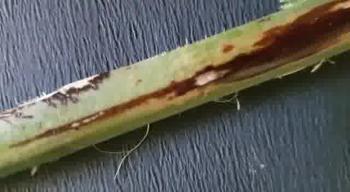

Supplement: S1 Data — (ZIP) [file pone.0282250.s001.zip › DatePalmData/Brown Spots/brownspots-120.jpg]

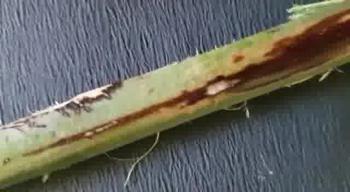

Supplement: S1 Data — (ZIP) [file pone.0282250.s001.zip › DatePalmData/Brown Spots/brownspots-121.jpg]

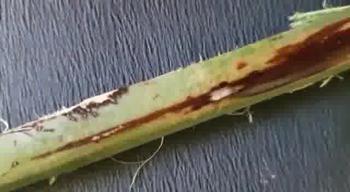

Supplement: S1 Data — (ZIP) [file pone.0282250.s001.zip › DatePalmData/Brown Spots/brownspots-122.jpg]

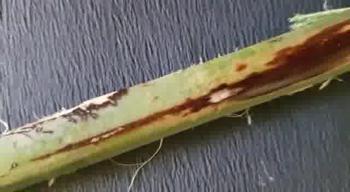

Supplement: S1 Data — (ZIP) [file pone.0282250.s001.zip › DatePalmData/Brown Spots/brownspots-123.jpg]

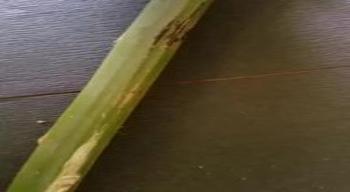

Supplement: S1 Data — (ZIP) [file pone.0282250.s001.zip › DatePalmData/Brown Spots/brownspots-124.jpg]

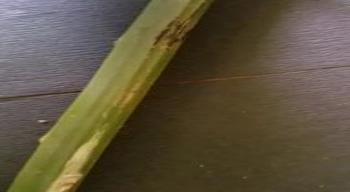

Supplement: S1 Data — (ZIP) [file pone.0282250.s001.zip › DatePalmData/Brown Spots/brownspots-125.jpg]

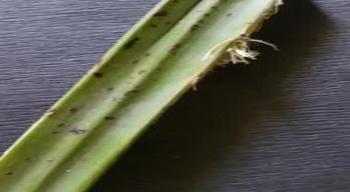

Supplement: S1 Data — (ZIP) [file pone.0282250.s001.zip › DatePalmData/Brown Spots/brownspots-126.jpg]

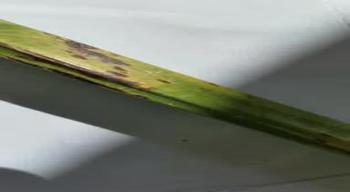

Supplement: S1 Data — (ZIP) [file pone.0282250.s001.zip › DatePalmData/Brown Spots/brownspots-127.jpg]

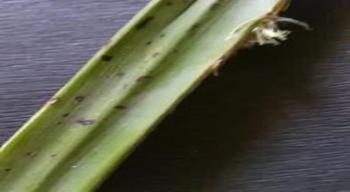

Supplement: S1 Data — (ZIP) [file pone.0282250.s001.zip › DatePalmData/Brown Spots/brownspots-128.jpg]

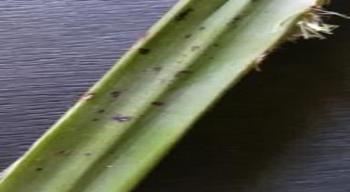

Supplement: S1 Data — (ZIP) [file pone.0282250.s001.zip › DatePalmData/Brown Spots/brownspots-129.jpg]

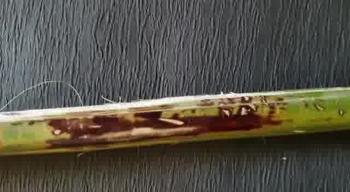

Supplement: S1 Data — (ZIP) [file pone.0282250.s001.zip › DatePalmData/Brown Spots/brownspots-13.jpg]

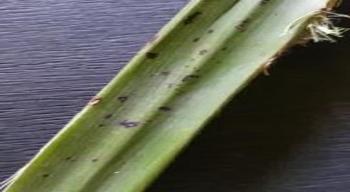

Supplement: S1 Data — (ZIP) [file pone.0282250.s001.zip › DatePalmData/Brown Spots/brownspots-130.jpg]

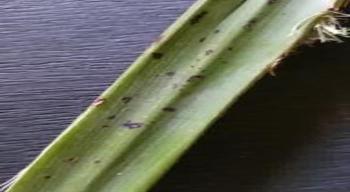

Supplement: S1 Data — (ZIP) [file pone.0282250.s001.zip › DatePalmData/Brown Spots/brownspots-131.jpg]

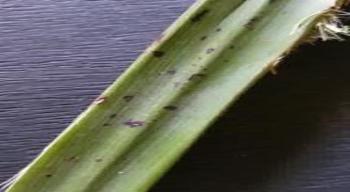

Supplement: S1 Data — (ZIP) [file pone.0282250.s001.zip › DatePalmData/Brown Spots/brownspots-132.jpg]

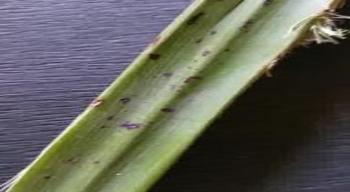

Supplement: S1 Data — (ZIP) [file pone.0282250.s001.zip › DatePalmData/Brown Spots/brownspots-133.jpg]

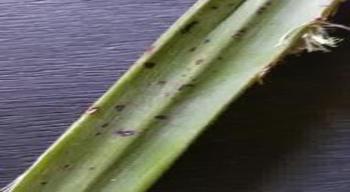

Supplement: S1 Data — (ZIP) [file pone.0282250.s001.zip › DatePalmData/Brown Spots/brownspots-134.jpg]

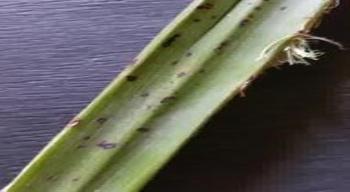

Supplement: S1 Data — (ZIP) [file pone.0282250.s001.zip › DatePalmData/Brown Spots/brownspots-135.jpg]

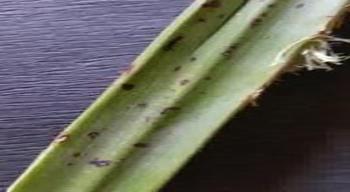

Supplement: S1 Data — (ZIP) [file pone.0282250.s001.zip › DatePalmData/Brown Spots/brownspots-136.jpg]

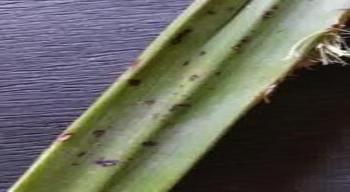

Supplement: S1 Data — (ZIP) [file pone.0282250.s001.zip › DatePalmData/Brown Spots/brownspots-137.jpg]

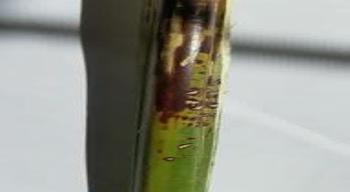

Supplement: S1 Data — (ZIP) [file pone.0282250.s001.zip › DatePalmData/Brown Spots/brownspots-138.jpg]

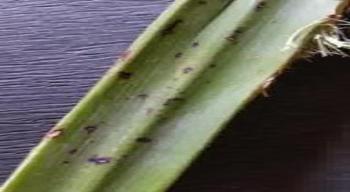

Supplement: S1 Data — (ZIP) [file pone.0282250.s001.zip › DatePalmData/Brown Spots/brownspots-139.jpg]

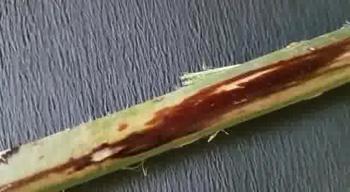

Supplement: S1 Data — (ZIP) [file pone.0282250.s001.zip › DatePalmData/Brown Spots/brownspots-14.jpg]

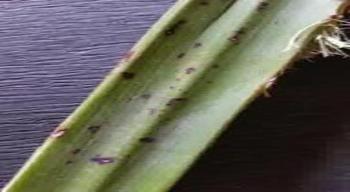

Supplement: S1 Data — (ZIP) [file pone.0282250.s001.zip › DatePalmData/Brown Spots/brownspots-140.jpg]

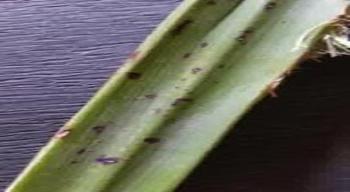

Supplement: S1 Data — (ZIP) [file pone.0282250.s001.zip › DatePalmData/Brown Spots/brownspots-141.jpg]

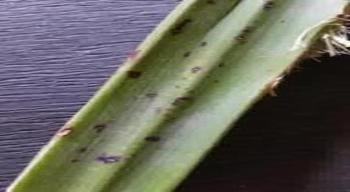

Supplement: S1 Data — (ZIP) [file pone.0282250.s001.zip › DatePalmData/Brown Spots/brownspots-142.jpg]

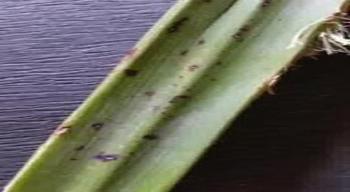

Supplement: S1 Data — (ZIP) [file pone.0282250.s001.zip › DatePalmData/Brown Spots/brownspots-143.jpg]

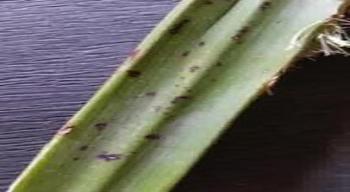

Supplement: S1 Data — (ZIP) [file pone.0282250.s001.zip › DatePalmData/Brown Spots/brownspots-144.jpg]

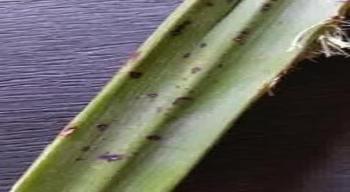

Supplement: S1 Data — (ZIP) [file pone.0282250.s001.zip › DatePalmData/Brown Spots/brownspots-145.jpg]

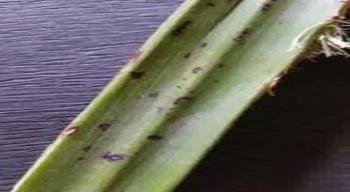

Supplement: S1 Data — (ZIP) [file pone.0282250.s001.zip › DatePalmData/Brown Spots/brownspots-146.jpg]

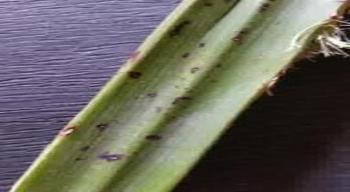

Supplement: S1 Data — (ZIP) [file pone.0282250.s001.zip › DatePalmData/Brown Spots/brownspots-147.jpg]

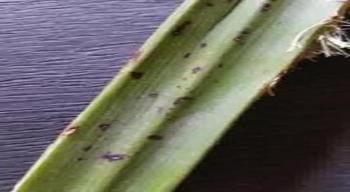

Supplement: S1 Data — (ZIP) [file pone.0282250.s001.zip › DatePalmData/Brown Spots/brownspots-148.jpg]

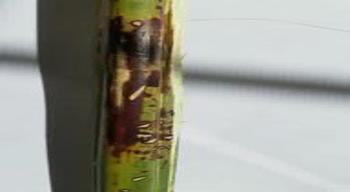

Supplement: S1 Data — (ZIP) [file pone.0282250.s001.zip › DatePalmData/Brown Spots/brownspots-149.jpg]

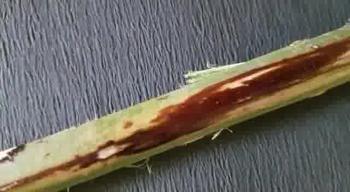

Supplement: S1 Data — (ZIP) [file pone.0282250.s001.zip › DatePalmData/Brown Spots/brownspots-15.jpg]

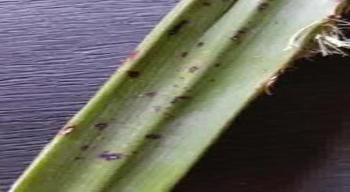

Supplement: S1 Data — (ZIP) [file pone.0282250.s001.zip › DatePalmData/Brown Spots/brownspots-150.jpg]

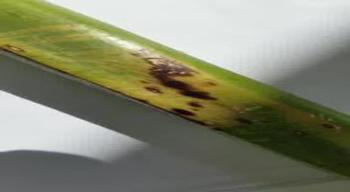

Supplement: S1 Data — (ZIP) [file pone.0282250.s001.zip › DatePalmData/Brown Spots/brownspots-151.jpg]

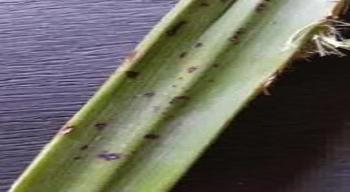

Supplement: S1 Data — (ZIP) [file pone.0282250.s001.zip › DatePalmData/Brown Spots/brownspots-152.jpg]

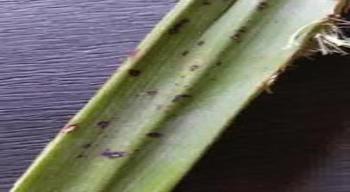

Supplement: S1 Data — (ZIP) [file pone.0282250.s001.zip › DatePalmData/Brown Spots/brownspots-153.jpg]

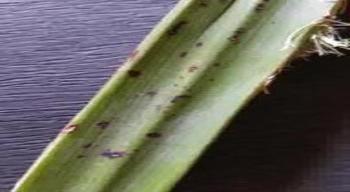

Supplement: S1 Data — (ZIP) [file pone.0282250.s001.zip › DatePalmData/Brown Spots/brownspots-154.jpg]

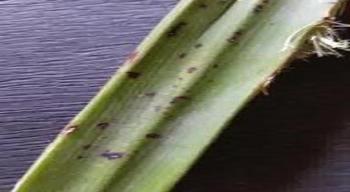

Supplement: S1 Data — (ZIP) [file pone.0282250.s001.zip › DatePalmData/Brown Spots/brownspots-155.jpg]

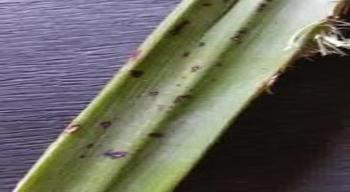

Supplement: S1 Data — (ZIP) [file pone.0282250.s001.zip › DatePalmData/Brown Spots/brownspots-156.jpg]

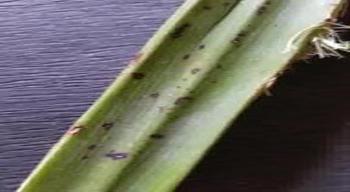

Supplement: S1 Data — (ZIP) [file pone.0282250.s001.zip › DatePalmData/Brown Spots/brownspots-157.jpg]

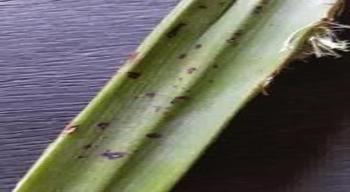

Supplement: S1 Data — (ZIP) [file pone.0282250.s001.zip › DatePalmData/Brown Spots/brownspots-158.jpg]

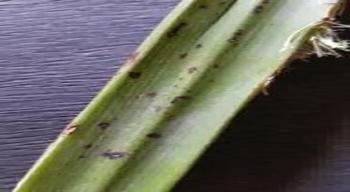

Supplement: S1 Data — (ZIP) [file pone.0282250.s001.zip › DatePalmData/Brown Spots/brownspots-159.jpg]

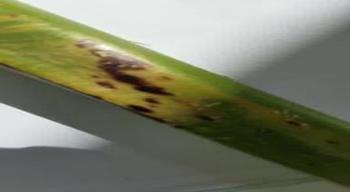

Supplement: S1 Data — (ZIP) [file pone.0282250.s001.zip › DatePalmData/Brown Spots/brownspots-16.jpg]

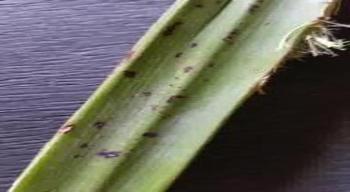

Supplement: S1 Data — (ZIP) [file pone.0282250.s001.zip › DatePalmData/Brown Spots/brownspots-160.jpg]

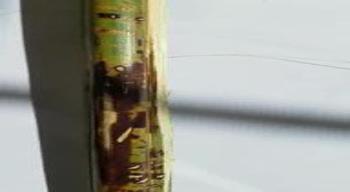

Supplement: S1 Data — (ZIP) [file pone.0282250.s001.zip › DatePalmData/Brown Spots/brownspots-161.jpg]

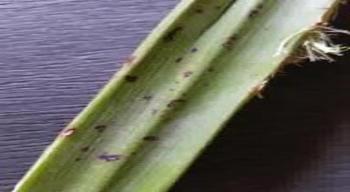

Supplement: S1 Data — (ZIP) [file pone.0282250.s001.zip › DatePalmData/Brown Spots/brownspots-162.jpg]

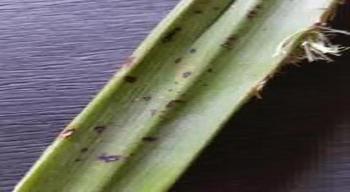

Supplement: S1 Data — (ZIP) [file pone.0282250.s001.zip › DatePalmData/Brown Spots/brownspots-163.jpg]

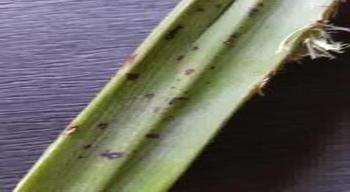

Supplement: S1 Data — (ZIP) [file pone.0282250.s001.zip › DatePalmData/Brown Spots/brownspots-164.jpg]

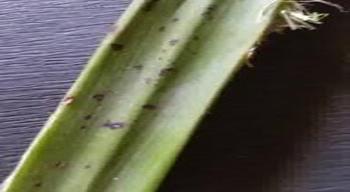

Supplement: S1 Data — (ZIP) [file pone.0282250.s001.zip › DatePalmData/Brown Spots/brownspots-165.jpg]

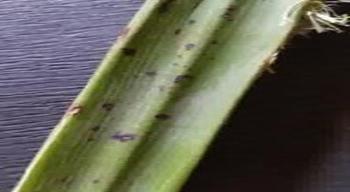

Supplement: S1 Data — (ZIP) [file pone.0282250.s001.zip › DatePalmData/Brown Spots/brownspots-166.jpg]

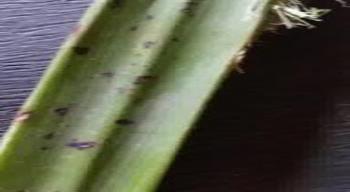

Supplement: S1 Data — (ZIP) [file pone.0282250.s001.zip › DatePalmData/Brown Spots/brownspots-167.jpg]

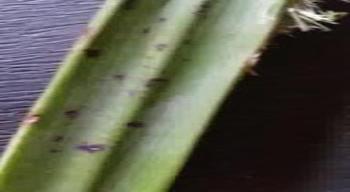

Supplement: S1 Data — (ZIP) [file pone.0282250.s001.zip › DatePalmData/Brown Spots/brownspots-168.jpg]

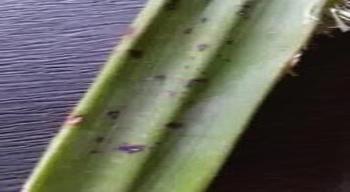

Supplement: S1 Data — (ZIP) [file pone.0282250.s001.zip › DatePalmData/Brown Spots/brownspots-169.jpg]

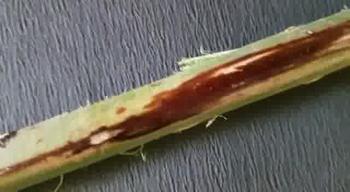

Supplement: S1 Data — (ZIP) [file pone.0282250.s001.zip › DatePalmData/Brown Spots/brownspots-17.jpg]

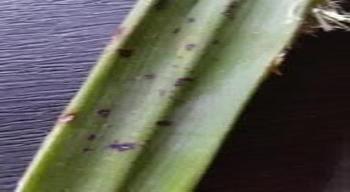

Supplement: S1 Data — (ZIP) [file pone.0282250.s001.zip › DatePalmData/Brown Spots/brownspots-170.jpg]

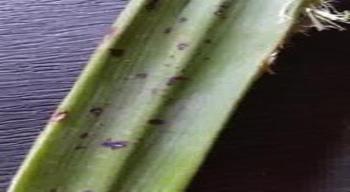

Supplement: S1 Data — (ZIP) [file pone.0282250.s001.zip › DatePalmData/Brown Spots/brownspots-171.jpg]

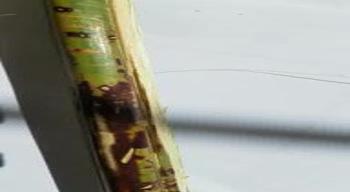

Supplement: S1 Data — (ZIP) [file pone.0282250.s001.zip › DatePalmData/Brown Spots/brownspots-172.jpg]

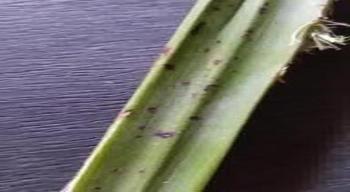

Supplement: S1 Data — (ZIP) [file pone.0282250.s001.zip › DatePalmData/Brown Spots/brownspots-173.jpg]

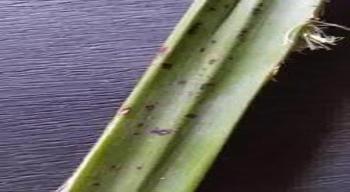

Supplement: S1 Data — (ZIP) [file pone.0282250.s001.zip › DatePalmData/Brown Spots/brownspots-174.jpg]

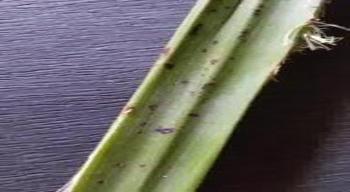

Supplement: S1 Data — (ZIP) [file pone.0282250.s001.zip › DatePalmData/Brown Spots/brownspots-175.jpg]

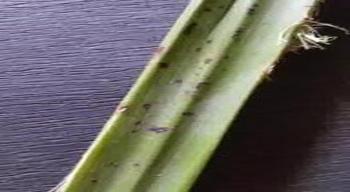

Supplement: S1 Data — (ZIP) [file pone.0282250.s001.zip › DatePalmData/Brown Spots/brownspots-176.jpg]

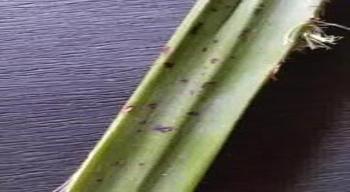

Supplement: S1 Data — (ZIP) [file pone.0282250.s001.zip › DatePalmData/Brown Spots/brownspots-177.jpg]

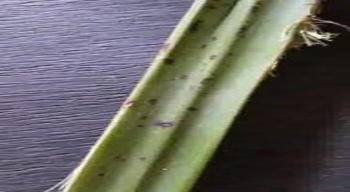

Supplement: S1 Data — (ZIP) [file pone.0282250.s001.zip › DatePalmData/Brown Spots/brownspots-178.jpg]

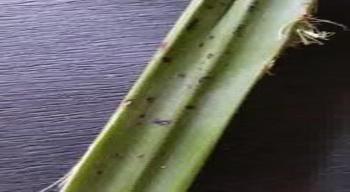

Supplement: S1 Data — (ZIP) [file pone.0282250.s001.zip › DatePalmData/Brown Spots/brownspots-179.jpg]

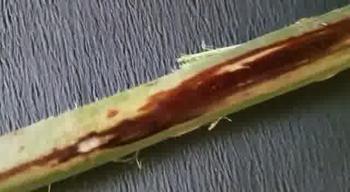

Supplement: S1 Data — (ZIP) [file pone.0282250.s001.zip › DatePalmData/Brown Spots/brownspots-18.jpg]

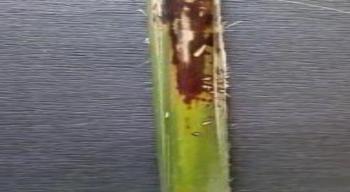

Supplement: S1 Data — (ZIP) [file pone.0282250.s001.zip › DatePalmData/Brown Spots/brownspots-180.jpg]

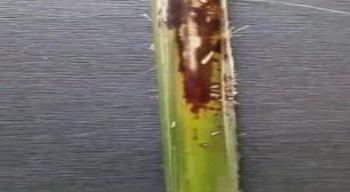

Supplement: S1 Data — (ZIP) [file pone.0282250.s001.zip › DatePalmData/Brown Spots/brownspots-181.jpg]

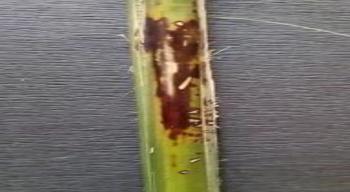

Supplement: S1 Data — (ZIP) [file pone.0282250.s001.zip › DatePalmData/Brown Spots/brownspots-182.jpg]

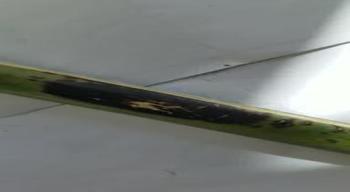

Supplement: S1 Data — (ZIP) [file pone.0282250.s001.zip › DatePalmData/Brown Spots/brownspots-183.jpg]

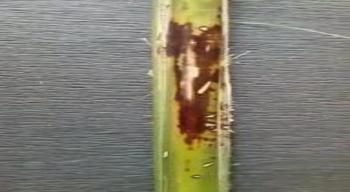

Supplement: S1 Data — (ZIP) [file pone.0282250.s001.zip › DatePalmData/Brown Spots/brownspots-184.jpg]

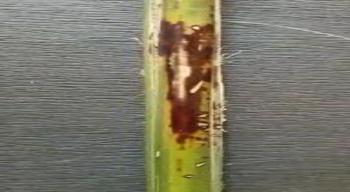

Supplement: S1 Data — (ZIP) [file pone.0282250.s001.zip › DatePalmData/Brown Spots/brownspots-185.jpg]

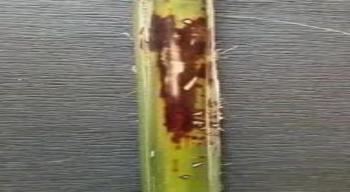

Supplement: S1 Data — (ZIP) [file pone.0282250.s001.zip › DatePalmData/Brown Spots/brownspots-186.jpg]

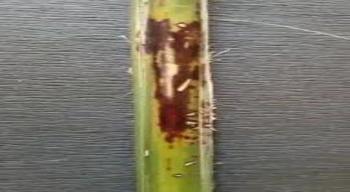

Supplement: S1 Data — (ZIP) [file pone.0282250.s001.zip › DatePalmData/Brown Spots/brownspots-187.jpg]

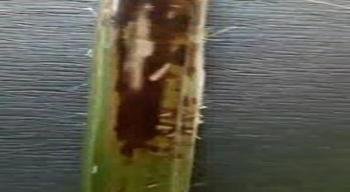

Supplement: S1 Data — (ZIP) [file pone.0282250.s001.zip › DatePalmData/Brown Spots/brownspots-188.jpg]

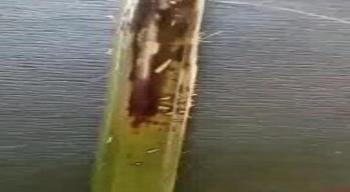

Supplement: S1 Data — (ZIP) [file pone.0282250.s001.zip › DatePalmData/Brown Spots/brownspots-189.jpg]
